# Synthetic chitin oligosaccharide nanocrystals and their higher-order assemblies†

**DOI:** 10.1039/d4sc07549h

**Published:** 2024-12-13

**Authors:** Surusch Djalali, Yun Jing, Yu Ogawa, Martina Delbianco

**Affiliations:** a Department of Biomolecular Systems, Max Planck Institute of Colloids and Interfaces Am Mühlenberg 1 Potsdam 14476 Germany martina.delbianco@mpukg.mpg.de; b Department of Chemistry and Biochemistry, Freie Universität Berlin Arnimallee 22 Berlin 14195 Germany; c Molecular Vista Inc. 6840 Via Del Oro, Suite 110 San Jose CA 95119 USA; d Univ. Grenoble Alpes, CNRS, CERMAV Grenoble 38000 France Yu.Ogawa@mpikg.mpg.de

## Abstract

Self-assembly is a powerful strategy for creating complex architectures and elucidating the aggregation behaviors of biopolymers. Herein, we investigate the hierarchical assembly of chitin using a *bottom-up* approach based on synthetic oligosaccharides. We discovered that chitin oligosaccharides self-assemble into platelets, which then form higher-order structures. Subtle changes in experimental conditions drastically altered the self-assembly results, generating a wide array of higher-order architectures. Through systematic investigations employing transmission electron microscopy (TEM), photoinduced force microscopy (PiFM), and atomic force microscopy (AFM), we uncovered the role of water in shaping the different morphologies. This finding gave us the tools to promote the formation of chiral, uniform chitin oligosaccharide bundles. Our work not only sheds light on the fundamental aspects of chitin organization, but also suggests strategies for designing carbohydrate-based materials with tunable structures and properties.

## Abstract 2

Chitin oligosaccharides self-assemble into platelets, which then further aggregate into chiral bundles.

## Graphical abstract

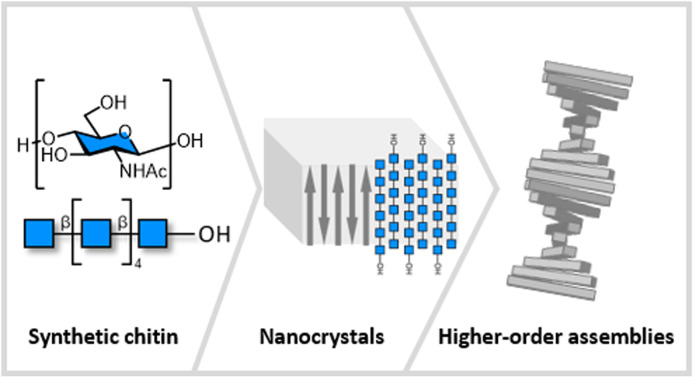

## Introduction

Studying the self-assembly of oligomer models is a powerful approach to understand the intricate rules governing biopolymer aggregation. This strategy has advanced our knowledge of molecular biology, particularly in the study of peptides^1–3^ and amyloids,^4–6^ revealing how specific sequences and structural features influence aggregation. In this context, not only the chemical structure but also experimental conditions, such as temperature, pH, and ionic strength, have been shown to affect the aggregation of peptide-based systems.^7–11^ Subtle changes of these parameters could shift the equilibrium between different morphologies, leading to the formation of distinct nanostructures, such as fibers, spheres, or even more complex hierarchical assemblies.^12,13^ Similarly, small differences in local solvent polarity can drastically influence peptide conformations and their subsequent assembly behavior.^14^ While this could be a severe source of irreproducibility,^11^ it also highlights the versatility of self-assembly approaches that, if controlled, could generate multiple architectures.^15,16^

Subtle changes in experimental conditions could have an even more dramatic effect for glycans, inherently flexible biomolecules adopting multiple conformations separated by low energy barriers.^17,18^ These polymers are known to aggregate into different morphologies,^19^ but the underlying mechanisms that drive the formation of a particular geometry are often unknown.^20^ A prominent example is chitin,^21,22^ an abundant polysaccharide of *N*-acetylglucosamine. This biopolymer can assemble into diverse crystalline morphologies,^23,24^ making chitin integral to the mechanical and structural properties of various biological entities, from arthropod exoskeletons to fungal cell walls.^25,26^ Still, a clear understanding of how chitin chemical structure (*e.g.* degree of polymerization, degree and pattern of acetylation) and environmental conditions influence these morphological features is lacking.^25^ Similarly, the chirality of chitin nanocrystals and their assemblies has been a subject of debate, following the observation that the chirality can vary depending on the specific treatment and processing conditions applied.^27,28^

Herein, we examine the different levels of chitin assembly following a *bottom up* approach that eliminates issues connected to chemical and structural heterogeneity of natural chitin. In contrast to existing *top down* chitin studies,^27,29^ our approach allows us to directly correlate morphological features of the assemblies to the molecular structure. We show that well-defined synthetic chitin oligosaccharides self-assemble into platelets that further aggregate into higher-order assemblies of varying morphologies. Subtle changes in environmental conditions dramatically altered the morphology of chitin-based architectures, offering a tool to produce well-defined chiral materials.

## Results and discussion

As model for our investigation, we synthesized a chitin hexasaccharide N_6_ by automated glycan assembly (AGA) (details for the synthesis are reported in the ESI†).^30^ This uniform oligomer, with well-defined chemical structure, offers an advantage against naturally sourced materials, avoiding issues of purity, batch to batch variability and ill-defined chemical structure. Powder X-ray diffraction (XRD) revealed that N_6_ assembled in the α-chitin crystal structure (Fig. S2†), the most prevalent allomorph of chitin in nature.^31^ Having confirmed that N_6_ preserved the same molecular assembly behavior (*i.e.* same crystal allomorph) of natural chitin, we set to investigate N_6_ aggregation behavior across multiple scales using transmission electron microscopy (TEM) and atomic force microscopy (AFM).

**Fig. 1:**
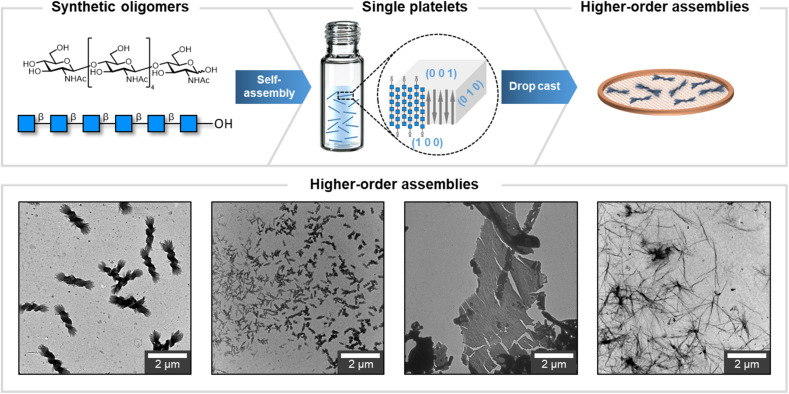
The multiple levels of chitin oligosaccharides assembly. Chitin oligomers (N_6_) assemble into a colloidally stable suspension of platelets with a 3-D molecular model adopting the α-chitin crystal structure. Drop casting generates different higher-order assemblies. TEM images of different types of higher-order assemblies obtained in this work. The morphologies range from defined twists, to less defined ones, to cramped material and to long, thin fibrils.

**Fig. 2:**
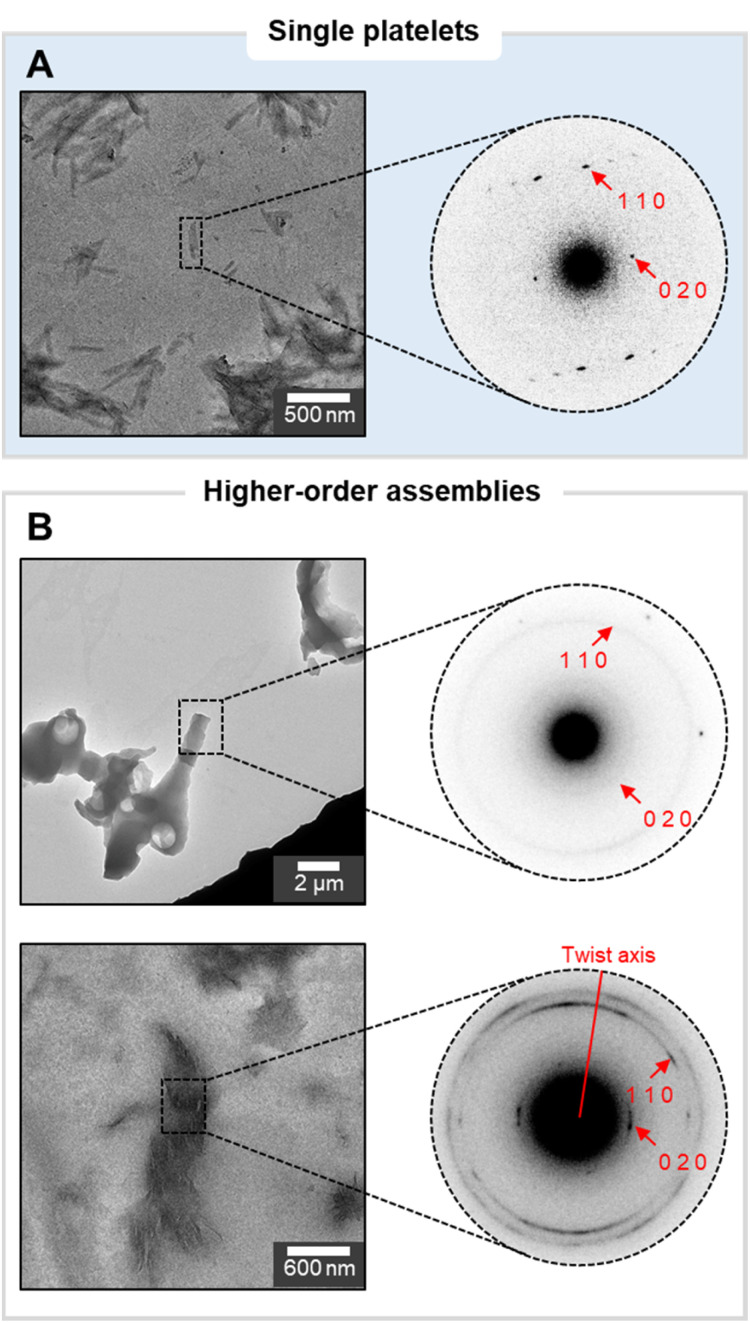
Electron diffraction pattern of a single platelet as reference (A) and of different higher-order assemblies (B). The sharpness of the reference peaks indicates a well-crystallized material with minimal internal strain and a uniform orientation of the oligomers along the crystal. The ring-pattern of the aggregates suggests an identical packing of the N_6_ chains, but with a random orientation along the aggregate. The twisted morphology shows the same interplanar spacing in the crystal structure as the reference but with a defined rotation of the pattern around the twist axis.

Previous studies on synthetic cellulose oligosaccharides revealed their tendency to self-assemble in aqueous solution into platelets.^32^ The higher solubility of N_6_ in water prevented aggregation at the concentration used for this study (up to 1 mg mL^−1^ in water).^33^ A change of solvent to the less polar isopropanol (i-PrOH) allowed us to visualize colloidally stable platelets of N_6_ oligomers (Fig. 1), as confirmed by cryo-TEM (Fig. S21†). Electron diffraction (ED) of the platelets confirmed their α-chitin-type molecular packing (Fig. 2A) and indicated the antiparallel alignment of oligomer chains along the platelet's thickness, with the (001) faces exposed at the top and bottom of the platelet. In polar environment, the (100) face presenting the hydrophobic GlcNAc face promoted crystal growth along the [100] direction, leading to the rod-like morphology (Fig. S20†).

Upon drop casting on a TEM grid, the N_6_ platelets suspension in i-PrOH generated a variety of higher-order assemblies with morphologies ranging from chiral twists (Fig. S13†), to less defined bundles, to thin fibrils (Fig. 1). These results highlighted severe issues of reproducibility (Fig. S14†), but also suggested the versatility of chitin-based materials to access a broad spectrum of distinct morphologies.

Thus, we set to investigate the underling factors that drive self-assembly of chitin oligomers. We reasoned that identifying the rules of chitin oligomers assembly will allow us to direct the formation of a particular morphology on demand.

We first screened the assembling tendency of oligomers with different degree of polymerization (DP). Similar higher-order morphologies were obtained upon drop casting a suspension of longer oligomers (N_7_) (Fig. S11†). In contrast, shorter oligomers (N_4_) proved too soluble, resulting in amorphous aggregates upon drying (Fig. S10 and S12†).

Then, we turned our attention to experimental conditions that could influence chitin oligomers assembly, using N_6_ as model system. In our experiments, the sample preparation procedure included the dilution of a stock solution of N_6_ (1 mg mL^−1^ in i-PrOH) to a target concentration of 0.1 to 0.5 mg mL^−1^ followed by sonication and drop casting of a 5 μL of suspension on a glow-discharged carbon-coated copper TEM grid (additional details in ESI†). The grid was left to dry for 1 h before imaging. Systematic variations of these parameters (Fig. S15†) as well as monitoring of sample aging (Fig. S16†) did not clarify the different results. Careful analysis of multiple experiments revealed a correlation with the date of the experiment, rather than with the experimental conditions.

Different higher-order assemblies were analyzed with ED to identify possible correlations between the molecular organization within the assemblies and their contrasting morphologies (Fig. 2). The analysis revealed that the crystal structure of the N_6_ oligomers within the platelets was consistent between all samples. In contrast, we identified substantial differences in the orientation of the platelets within the higher-order assemblies. While in some cases the platelets were randomly oriented, in other samples the single platelets aligned along the assembly axes, generating twisted bundles (Fig. 2B). These observations identified the drop casting process (*i.e.* formation of higher-order assemblies upon evaporation) rather than the initial assembly step (*i.e.* crystallization into platelets in solution) as the cause of irreproducible results.

**Fig. 3:**
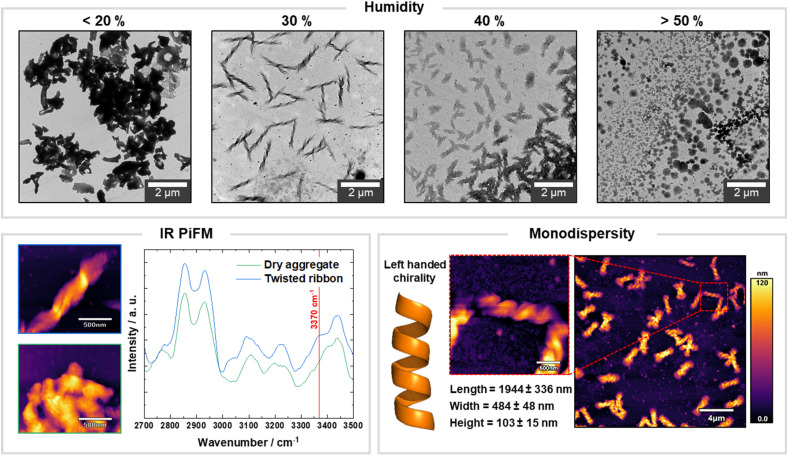
The morphology of synthetic chitin oligosaccharide assemblies is affected by humidity. TEM images showing that 30% of relative humidity favors the formation of chiral bundles. PiF-IR spectra with the expected absorbance peaks of chitin. The highlighted shoulder peak at 3370 cm^−1^ supports the hypothesis that water becomes an essential part of assembly, helping to orient the platelets with a regular displacement. Under controlled humidity, uniform left-handed chiral bundled can be obtained as shown by AFM analysis.

We then analyzed potential environmental factors that could affect the drop casting step. While the laboratory had a stable temperature (25 ± 2 °C), we noticed severe fluctuations in the humidity (relative humidity 1 to 60%) depending on the season and daytime. To prove the hypothesis that the humidity in the air influenced the morphology of the higher-order assemblies, we drop casted the sample under controlled humidity. A humidifier, capable of creating humidified air with a precision of ±1%, connected to a sealed polyacrylic box allowed us to spot our samples under controlled atmosphere (see ESI† for details of the set up). With this set up, we demonstrated that in dry conditions (<20%) the single platelets randomly oriented, forming heterogenous non-defined assemblies (Fig. 3). At 30% humidity, the morphology of the assembly shifted to well-defined chiral bundles (Fig. 3). At higher humidity (>40%), the higher amount of water that diffused into the i-PrOH suspension disrupted the formation of platelets. Therefore, only amorphous aggregates without crystalline features were obtained (Fig. 3).

The presence of water diffusing into i-PrOH seemed to guide the assembly of platelets, allowing for the formation of uniform bundles. Thus, we tested whether similar results could be obtained performing the assembly in an i-PrOH/H_2_O mixture (spotted under dry conditions, humidity 1%). Systematic variation of the water content revealed a similar trend to what observed under controlled humidity (Fig. S17†), highlighting the key role of water in dictating the morphology of higher-order assemblies. This realization allowed us to eliminate reproducibility issues during the formation of higher-order assemblies.

To dissect the role of water in the assembly process, we analyzed our samples with photoinduced force microscopy (PiFM, Fig. 3).^34^ This technique combines the imaging capability of an AFM with the chemical information of local infrared spectroscopy (IR), allowing to detect and localize different functional groups within the different types of aggregates with a sub 10 nm spatial resolution.^35^ We recorded IR spectra for the twisted bundles obtained at 30% humidity and for the aggregates generated at low humidity. Both spectra shared characteristic features of α-chitin, but showed a substantial difference in the O–H stretching region (Fig. 3).^36^ For the twisted bundles, an additional shoulder peak at around 3370 cm^−1^ occurred, suggesting that water was most likely within the bundle. The results supported the hypothesis that water is part of the structure and plays an active role in the formation of different types of morphologies. We speculate that water induces the organized self-assembly of the colloidally stable platelets, by influencing the thermodynamics of their aggregation process. Similar behavior was observed for peptide self-assemblies where water had a severe impact on the shape and morphology of a peptide system.^37^

Having identified the fundamental role of water in the assembly process gave us the tools to control and favor the formation of chiral uniform bundles. By performing the assembly in a humidity-controlled environment, we could generate exclusively left-handed twists with an average length of around 2 μm, a width of 500 nm and a height of 100 nm (Fig. 3, S21 and S22†). This result highlights the *bottom up* approach as a valuable strategy to obtain carbohydrate materials with controlled morphology and chirality. Moreover, when the samples prepared in dry conditions were incubated in high humidity conditions overnight, we could observe morphological changes, suggesting the potential applications of chitin based-materials in responsive devices (Fig. S18 and S19†).

## Conclusion

We showed that synthetic oligomers of chitin self-assembled into platelets that further aggregated into higher-order assemblies. Environmental humidity dramatically impacted the morphology of the higher-order assemblies, stressing the key role of water in tuning the morphology of chitin-based architectures.^38^ Variations in humidity resulted in a diverse range of morphologies, from heterogeneous, non-defined assemblies to well-defined chiral bundles. This variability, initially seen as source of irreproducible results, could be exploited to tune the self-assembly process and generate multiple architectures on demand. While this remains a simplified model, we argue that similar mechanisms could be involved in the aggregation of chitin structures in nature.^26^ Follow up studies will clarify how other parameters such as degree of polymerization, deacetylation (degree and pattern), and presence of additives can further direct this assembly behaviour.

The *bottom up* approach presented here improved our understanding of the fundamental principles governing chitin assembly and inspired new avenues for the development of carbohydrate materials with tailored properties. Following careful assembly procedures and systematic studies, we argue that glycan versatility could open up several opportunities for designing responsive materials.^39^ For example, the introduction of a controlled humidity protocol during sample preparation offered us a tool to manipulate the assembly behavior of chitin oligosaccharide platelets to generate chiral, uniform twists. This system could inspire the design of light-interacting materials^40,41^ or chiral catalysts.^42,43^

## Data availability

The data that support the findings of this study are available within the article and ESI.†

## Author contributions

S. D. designed and performed the experiment and wrote the manuscript. Y. O. perform the cryo-TEM and ED analysis with constructive discussion. Y. J. performed the PiFM experiments. M. D. and Y. O. supervised the project and wrote the manuscript. All authors have given approval to the final version of the manuscript.

## Conflicts of interest

The authors declare no competing interests.

## Supplementary Material

SC-OLF-D4SC07549H-s001
