# Supplementary material for: Synthetic chitin oligosaccharide nanocrystals and their higher-order assemblies
Source: Chem Sci. 2024 Dec 13;16(3):1390–5. doi: 10.1039/d4sc07549h (PMC11653566; doi:10.1039/d4sc07549h)
Supplement: SC-016-D4SC07549H-s001 [file SC-016-D4SC07549H-s001.pdf]

Supplementary Information (SI) for Chemical Science.
This journal is © The Royal Society of Chemistry 2024

Supporting Information

Synthetic chitin oligosaccharide nanocrystals and their higher-order assemblies

Surusch Djalali,[a,b] Yun Jing,[c] Yu Ogawa,[d*] and Martina Delbianco[a*]

[a] Department of Biomolecular Systems, Max Planck Institute of Colloids and Interfaces, Am Mühlenberg 1, 14476 Potsdam, Germany

[b] Department of Chemistry and Biochemistry, Freie Universität Berlin, Arnimallee 22, 14195 Berlin, Germany

[c] Molecular Vista Inc., 6840 Via Del Oro, Suite 110, San Jose, CA 95119, USA

[d] Univ. Grenoble Alpes, CNRS, CERMAV, 38000 Grenoble, France

Table of Contents

1 General Materials and Methods 3

2 Synthesis of Building Blocks 4

3 Automated Glycan Assembly 5

3.1 General materials and methods 5

3.2 Preparation of stock solutions 5

3.3 Modules for automated synthesis 5

3.4 Post-AGA manipulations 7

3.5 Synthesis of N_6 9

3.6 Comparison to commercial N_6 12

3.7 Synthesis of N_4 13

3.8 Synthesis of N_7 16

4 Oligosaccharides self-assembly 19

4.1 X-ray diffraction analysis 19

4.2 Transmission electron microscopy imaging 20

4.2.1 N_6 in H_2O 21

4.2.2 N_6 in hexafluoroisopropanol 21

4.2.3 N_6 in methanol 21

4.2.4 N_6 in ethanol 22

4.2.5 N_6 in isopropanol 22

4.2.6 Commercial N_6 in isopropanol 23

4.2.7 N_4 in isopropanol 23

4.2.8 N_7 in isopropanol 23

4.2.9 Degree of polymerization 24

4.2.10 N6 with controlled humidity 26

4.2.11 Cryo-TEM of N_6 in isopropanol 28

4.3 Atomic force microscopy imaging 29

4.4 Photo-induced force microscopy (PiFM) 30

5 References 31

1 General Materials and Methods

All chemicals used were reagent grade and used as supplied unless otherwise noted. The automated syntheses were performed on a home-built synthesizer developed at the Max Planck Institute of Colloids and Interfaces.[1] Analysis and purification was performed by reverse phase HPLC was performed by using an Agilent 1200 series. Products were lyophilized using a Christ Alpha 2-4 LD plus freeze dryer. ^{1}H, ^{13}C and HSQC NMR spectra were recorded on a Varian 400-MR (400 MHz), Varian 600-MR (600 MHz), or Bruker Biospin AVANCE700 (700 MHz) spectrometer. Spectra were recorded in $CDCl_3$ by using the solvent residual peak chemical shift as the internal standard ($CDCl_3$: 7.26 ppm ^{1}H, 77.0 ppm ^{13}C) or in D_2O using the solvent as the internal standard in ^{1}H NMR (D_2O: 4.79 ppm ^{1}H). ^{1}H NMR integrals of the resonances corresponding to residues at the reducing end are reported as non-integer numbers and the sum of the integrals of α and β anomers is set to 1. High resolution mass spectra were obtained using a 6210 ESI-TOF mass spectrometer (Agilent) and a MALDI-TOF autoflex™ (Bruker). MALDI and ESI mass spectra were run on IonSpec Ultima instruments. IR spectra were recorded on a Perkin-Elmer 1600 FTIR spectrometer.

The synthesis of the synthetic chitin oligosaccharide N_6 was previously published as stated in the main text. We report the synthetic procedure and characterization of the chitin hexasaccharide for completion. The original reference can be found here [2].

2 Synthesis of Building Blocks

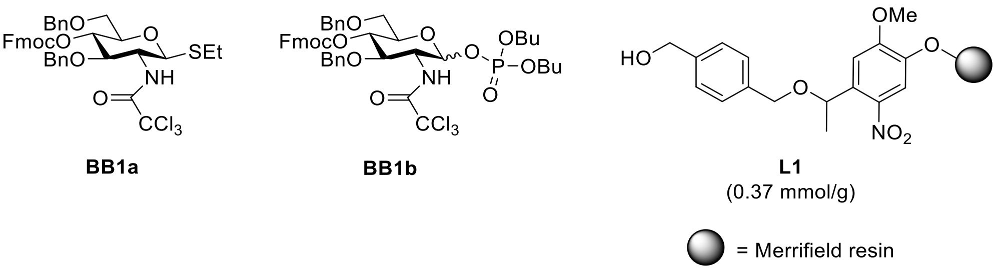

Figure S1 – BBs and solid supports used in this work.

Building block **BB1a** was purchased from GlycoUniverse (Germany). Building block **BB1b** (α:β = 18:1)was synthesized according to previously reported procedures.[2] Merrifield resin equipped with a photocleavable linker (**L1**, loading 0.35 mmol/g) was prepared according to previous literature.[3]

3 Automated Glycan Assembly

3.1 General materials and methods

The automated syntheses were performed on a home-built synthesizer developed at the Max Planck Institute of Colloids and Interfaces.[1] All solvents used were HPLC-grade. The solvents used for the building block, activator, TMSOTf and capping solutions were taken from an anhydrous solvent system (J.C. Meyer) for moisture-sensitive solutions. The building blocks were co-evaporated three times with toluene and dried for 1 h under high vacuum before use. Oven-heated, argon-flushed flasks were used to prepare all moisture-sensitive solutions. Activator, capping, deprotection, acidic wash and building block solutions were freshly prepared and kept under argon during the automation run. All yields of products obtained by AGA were calculated on the basis of resin loading. Resin loading was determined following previously established procedures.[4]

3.2 Preparation of stock solutions

- **Building block solution**: 0.06 mmol (5 equiv.) of glycosyl phosphate donor was dissolved in DCM (1 mL).
- **Fmoc deprotection solution**: A solution of 20% piperidine in DMF (v/v) was prepared.
- **TMSOTf solution**: TMSOTf (0.45 mL, 2.49 mmol) was added to DCM (40 mL).
- **Capping solution**: A solution of 10% acetic anhydride and 2% methanesulfunic acid in DCM (v/v) was prepared.

3.3 Modules for automated synthesis

Module A: Resin Preparation for Synthesis (20 min)

All automated syntheses were performed on 0.0125 mmol scale. Resin (**L1**, 34 mg) was placed in the reaction vessel and swollen in DCM for 20 min at room temperature prior to synthesis. During this time, all reagent lines needed for the synthesis were washed and primed. After the swelling, the resin was washed with DMF, THF, and DCM (three times each with 2 mL for 25 s).

Module B: Acidic Wash with TMSOTf Solution (20 min)

The resin was swollen in 2 mL DCM and the temperature of the reaction vessel was adjusted to -20 °C. Upon reaching the low temperature, TMSOTf solution (1 mL) was added drop wise to the reaction vessel. After bubbling for 3 min, the acidic solution was drained and the resin was washed with 2 mL DCM for 25 s.

Action	Cycles	Solution	Amount	T (°C)	Incubation time
**Cooling**	-	-	-	-20	(15 min)*
**Deliver**	1	DCM	2 mL	-20	-
**Deliver**	1	TMSOTf solution	1 mL	-20	3 min
**Wash**	1	DCM	2 mL	-20	25 sec

*Time required to reach the desired temperature.

Module C: Phosphate Glycosylation (45 min)

The building block solution (0.06 mmol of BB in 1 mL of DCM) was delivered to the reaction vessel. After the set temperature was reached, the reaction was started by drop wise addition of the TMSOTf solution (1.0 mL). After completion of the reaction, the solution was drained and the resin washed with DCM (six times, each with 2 mL for 25 s). The temperature of the reaction vessel was increased to 25 °C for the next module.

Action	Cycles	Solution	Amount	T (°C)	Incubation time
**Cooling**	-	-	-	T_1	(20 min)*
**Deliver**	1	BB solution	1 mL	T_1	-
**Deliver**	1	TMSOTf solution	1 mL	T_1	-
**Reaction time (BB dependent)**	1			T_1 to T_2	t_1 t_2 min
**Wash**	1	DCM	2 mL	T_2	5 sec
**Heating**	-	-	-	25	-
**Wash**	6	DCM	2 mL	> 0	25 sec

*Time required to reach the desired temperature.

The AGA glycosylation conditions employed for the phosphate BB were previously reported.[2]

BB	Equiv.	t_1 (min)	T_1 (°C)	t_2 (min)	T_2 (°C)
**BB1b**	5	5	-35	40	-15

Module D: Capping (30 min)

The resin was washed with DMF (two times with 2 mL for 25 s) and the temperature of the reaction vessel was adjusted to 25 °C. 2 mL of Pyridine solution (10% in DMF) was delivered into the reaction vessel. After 1 min, the reaction solution was drained and the resin washed with DCM (three times with 3 mL for 25 s). 4 mL of capping solution was delivered into the reaction vessel. After 20 min, the reaction solution was drained and the resin washed with DCM (three times with 3 mL for 25 s).

Action	Cycles	Solution	Amount	T (°C)	Incubation time
**Heating**	-	-	-	25	(5 min)*
**Wash**	2	DMF	2 mL	25	25 sec
**Deliver**	1	10% Pyridine in DMF	2 mL	25	1 min
**Wash**	3	DCM	2 mL	25	25 sec
**Deliver**	1	Capping Solution	4 mL	25	20 min
**Wash**	3	DCM	2 mL	25	25 sec

*Time required to reach the desired temperature.

Module E: Fmoc Deprotection (9 min)

The resin was washed with DMF (three times with 2 mL for 25 s) and the temperature of the reaction vessel was adjusted to 25 °C. 2 mL of Fmoc deprotection solution was delivered to the reaction vessel and kept under Ar bubbling. After 5 min, the reaction solution was drained and the resin washed with DMF (three times with 3 mL for 25 s) and DCM (five times each with 2 mL for 25 s). The temperature of the reaction vessel was decreased to -20 °C for the next module.

Action	Cycles	Solution	Amount	T (°C)	Incubation time
**Wash**	3	DMF	2 mL	25	25 sec
**Deliver**	1	Fmoc depr. Solution 1	2 mL	25	5 min
**Wash**	1	DMF	2 mL		
**Cooling**	-	-	-	-20	-
**Wash**	3	DMF	2 mL	< 25	25 sec
**Wash**	5	DCM	2 mL	< 25	25 sec

3.4 Post-AGA manipulations

Module F: Cleavage from Solid Support

The oligosaccharides were cleaved from the solid support using a continuous-flow photoreactor as described previously.[5]

Module H: Hydrogenolysis

The crude compound obtained from *Module F* was dissolved in 2 mL of EtOAc:*t*BuOH:H_2O (2:1:1). 100% by weight $Pd(OH)_2/C$ (10-20%) was added and the reaction was stirred in a pressurized reactor under 4 bar pressure of H_2. The reaction progress was monitored to avoid undesired side products formation (*i.e.* degradation of reducing end).[9] Upon completion, the reaction was filtered and washed with EtOAc, *t*BuOH and H_2O (4 mL each). The filtrates were concentrated *in vacuo.*

Module I: Purification

The purification of the crudes was conducted using a reverse phase HPLC (Agilent 1200 Series, **Method A2**). The pure compound was analyzed using analytical HPLC (Agilent 1200 Series, **Method A1**).

- **Method A1:** (Hypercarb column, 150 x 4.6 mm) flow rate of 0.7 mL / min with H_2O (0.1% formic acid) as eluents [isocratic (5 min), linear gradient to 40% ACN (30 min), linear gradient to 100% ACN (10 min)].
- **Method A2 (Prep):** (Hypercarb column, 150 x 10 mm) flow rate of 1.3 mL / min with H_2O (0.1% formic acid) as eluents [isocratic (5 min), linear gradient to 30% ACN (30 min), linear gradient to 100% ACN (5 min)].
- **Method A3:** (Hypercarb column, 150 x 4.6 mm) flow rate of 0.7 mL / min with H_2O (0.1% formic acid) as eluents [isocratic (5 min), linear gradient to 30% ACN (30 min), linear gradient to 100% ACN (10 min)].
- **Method A4:** (Hypercarb column, 150 x 4.6 mm) flow rate of 0.7 mL / min with H_2O (0.1% formic acid) as eluents [isocratic (5 min), linear gradient to 60% ACN (30 min), linear gradient to 100% ACN (10 min)].

Following final purification, all deprotected products were lyophilized on a Christ Alpha 2-4 LD plus freeze dryer prior to characterization.

3.5 Synthesis of N_6

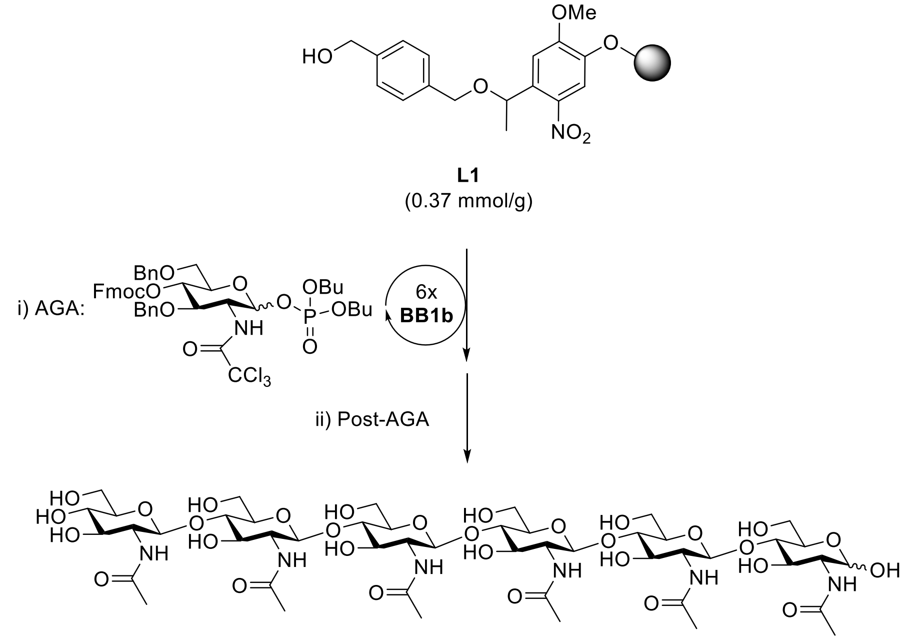

N_6

Step	Modules		Notes
AGA	**L1**<br>(**BB1b**)x6	**A**<br>(**B**, **C**, **D**, **E**)x6	**L1** swelling<br>**C**: (**BB1b**, -35 °C for 5 min, -10 °C for 40 min)
Post-AGA		**F**, **G**, **H**, **I**	**F**: (16 h)<br>**H**: (overnight)<br>**I**: (Method A2)

Automated synthesis, global deprotection, and purification afforded compound **N_6** as a white solid (1.4 mg, 8% overall yield).

Analytical data for **N_6**:
^{1}H NMR (600 MHz, D_2O) δ 5.40 – 5.38 (m, 1H), 5.21 (d, *J* = 2.8 Hz, 0.55H, α-H1), 4.71 (d, *J* = 8.2 Hz, 0.45H, β-H1), 4.60 (dd, *J* = 8.1, 5.4 Hz, 5H, internal H1), 3.99 – 3.45 (m, 39H), 2.00 (s, 3H, acetyl), 1.99 (s, 12H, acetyls), 1.97 (s, 3H, acetyl); ^{13}C NMR (151 MHz, D_2O) δ 174.53, 174.50, 174.37, 170.96, 101.37, 101.15, 94.74 (β-C1), 90.36 (α-C1), 79.54, 79.06, 78.83, 75.82, 74.49, 74.45, 74.42, 73.35, 72.04, 71.97, 69.91, 69.61, 69.15, 60.45, 59.92, 59.89, 59.85, 55.49, 54.98, 54.96, 54.96, 54.92, 54.92, 53.56, 22.08, 22.02; m/z (HRMS+) 1259.476 [M + Na]$^+$ ($C_{48}H_{81}N_6O_{31}Na$ requires 1259.477).

RP-HPLC of N_6 (ELSD trace, Method A1, t_R = 23.6 min: β-anomer, 24.5 min: α-anomer)

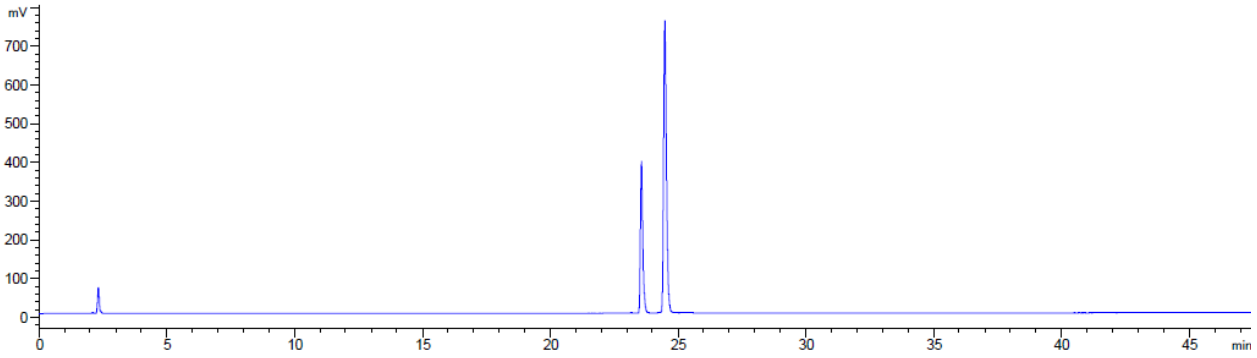

^1H NMR of N_6 (400 MHz, D_2O)

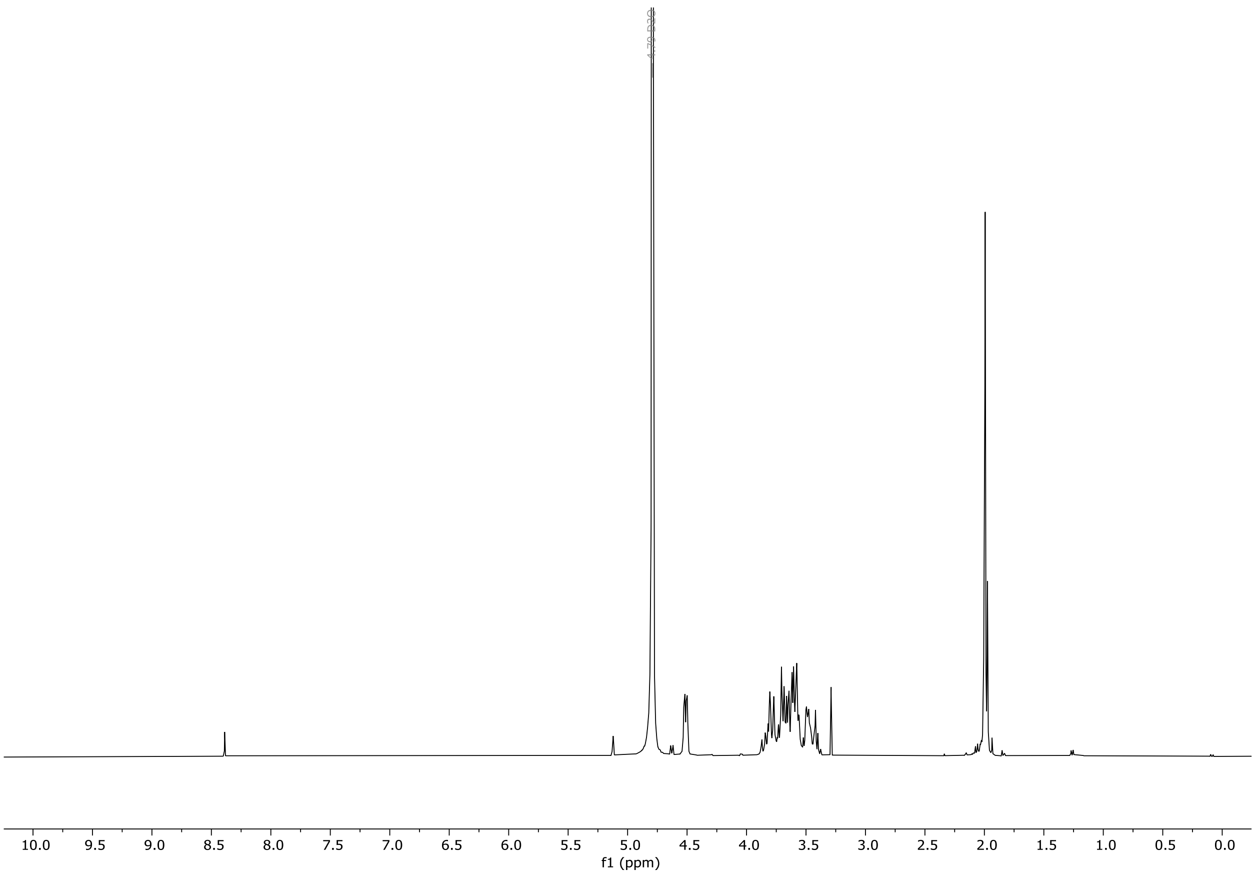

^{13}C NMR of N_6 (151 MHz, D_2O)

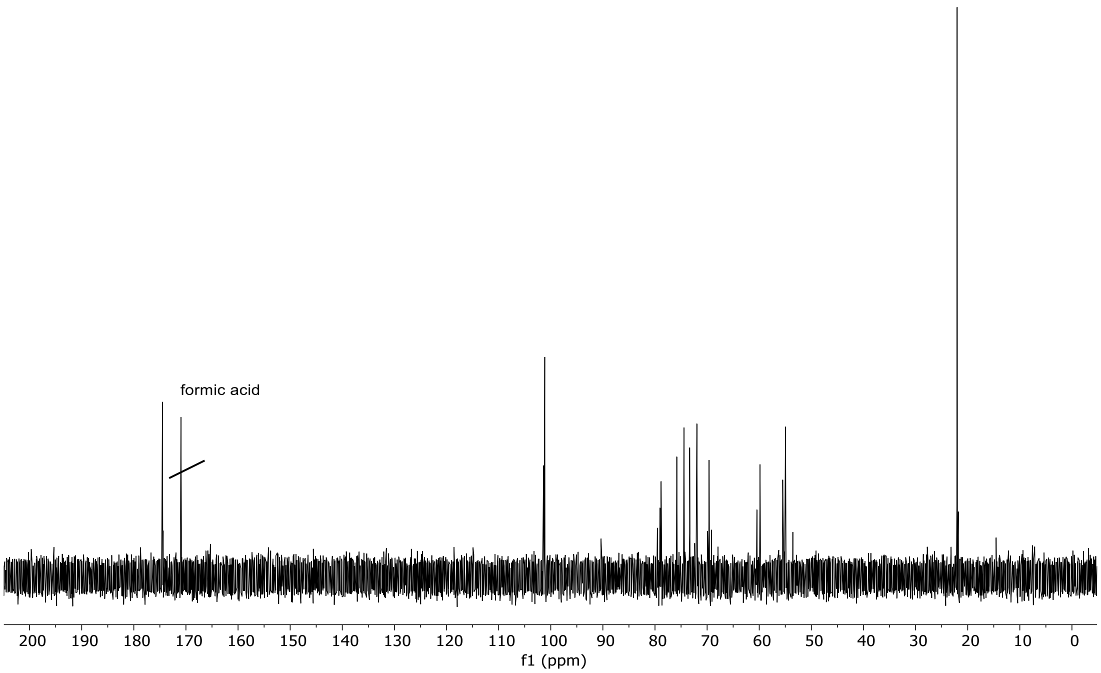

HSQC NMR of N_6 (D_2O)

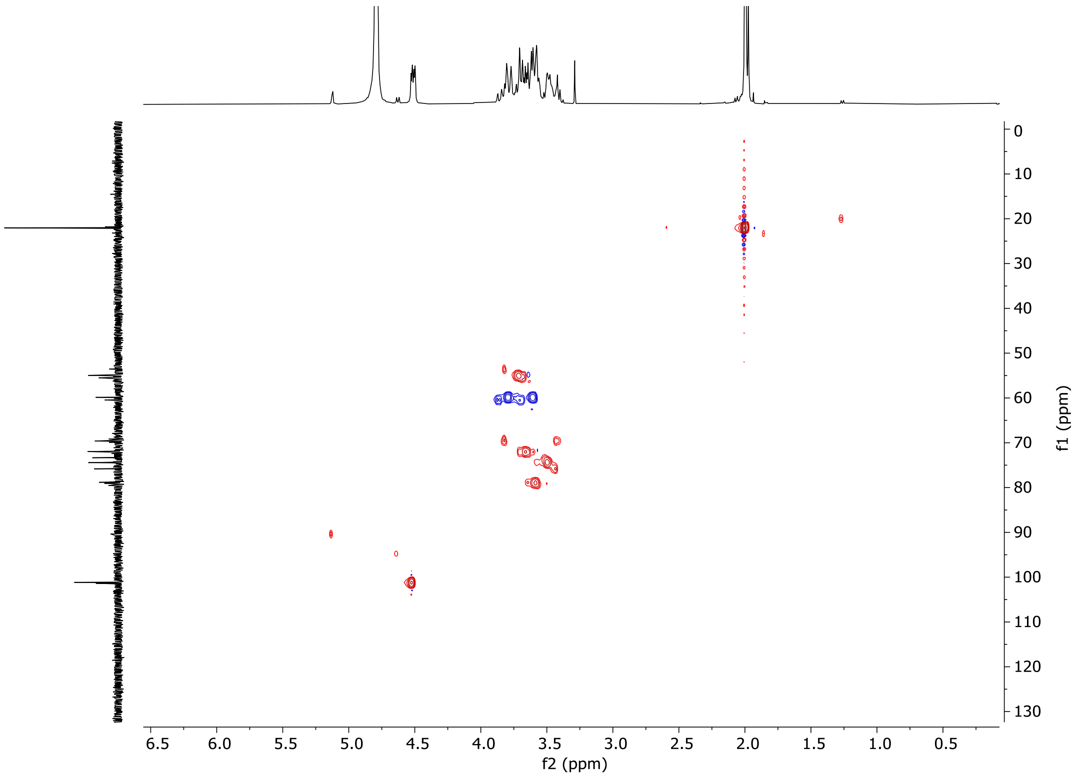

3.6 Comparison to commercial N_6

A commercially available sample of N_6, extracted and purified from natural precursors and sold by *Hycultec*, was purchased to compare the results with the fully synthetic N_6 synthesized in this work. The reported purity is 97.5%, as measured by HPLC. The following figure compares the ^{1}H NMR of both N_6 samples in D_2O.

^{1}H NMR of N_6 (400 MHz, D_2O)

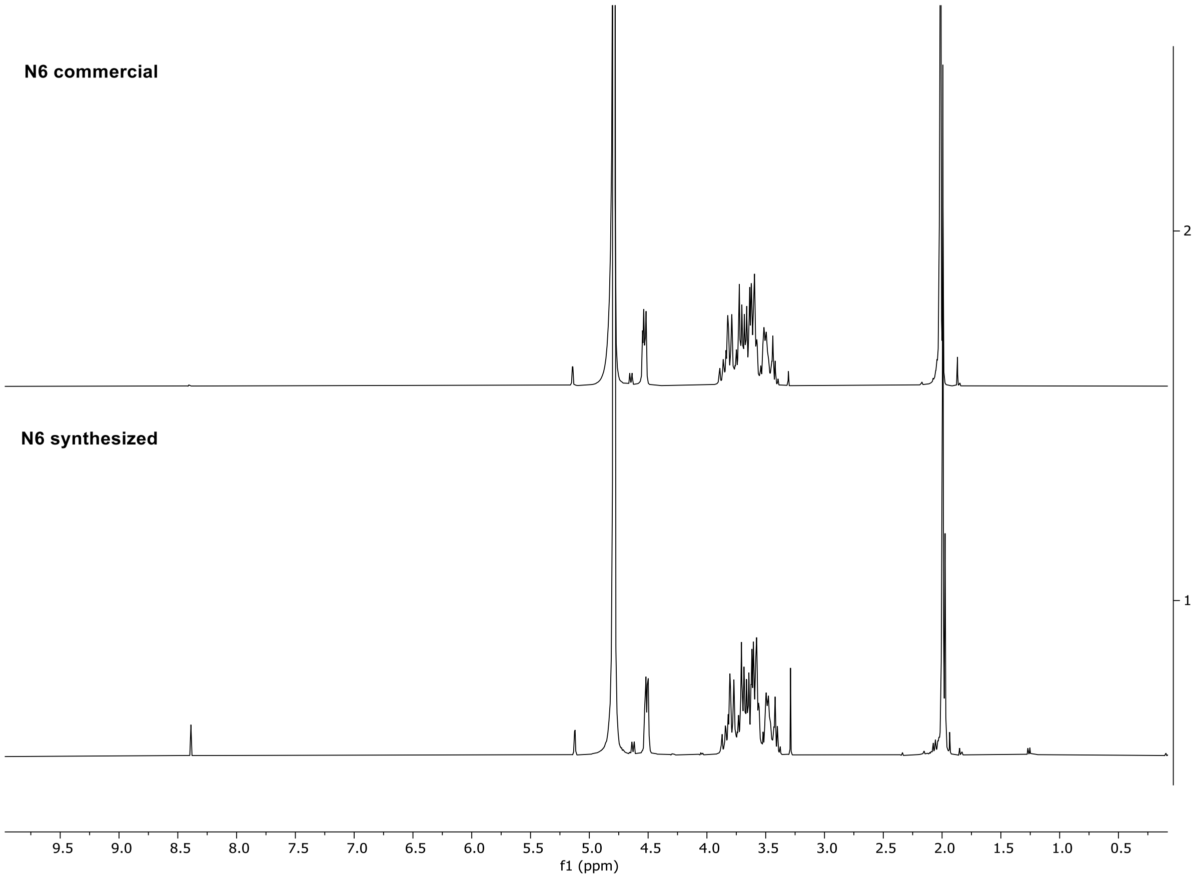

3.7 Synthesis of N_4

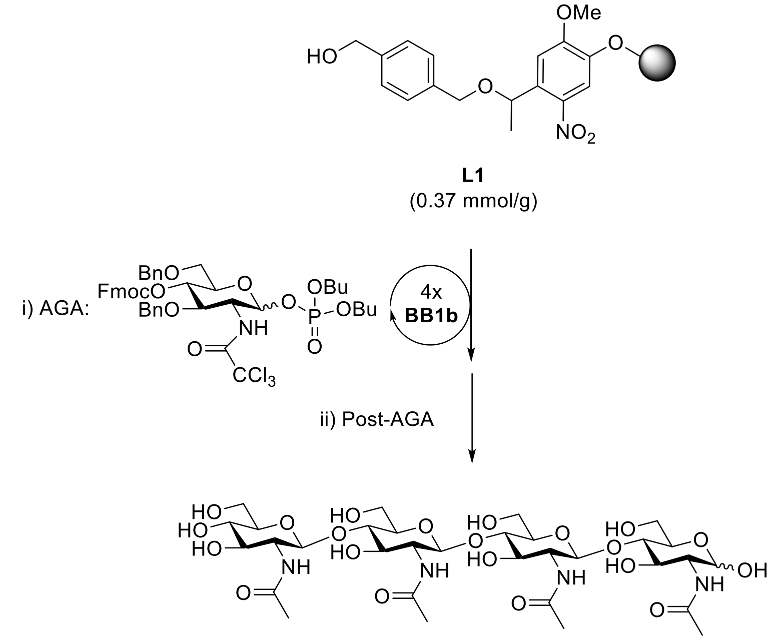

N_4

Step	Modules		Notes
AGA	**L1**	**A**	**L1** swelling
	(**BB1b**)x4	(**B**, **C**, **D**, **E**)x4	**C**: (**BB1b**, -35 °C for 5 min, -10 °C for 40 min)
Post-AGA		**F**, **G**, **H**, **I**	**F**: (16 h)
			**H**: (overnight)
			**I**: (Method A2)

Automated synthesis, global deprotection, and purification afforded compound **N_4** as a white solid (2.9 mg, 28% overall yield).

Analytical data for **N_4**: ^{1}H NMR (400 MHz, D_2O) δ 5.11 (d, *J* = 2.8 Hz, 0.6H, α-H1), 4.61 (d, *J* = 8.0 Hz, 0.4H, β-H1), 4.55 – 4.45 (m, 3H, internal H1), 3.88 – 3.75 (m, 5H), 3.75 – 3.52 (m, 14H), 3.52 – 3.35 (m, 6H), 1.99 (s, 3H, acetyl), 1.98 (s, 3H, acetyl), 1.98 (s, 3H, acetyl), 1.96 (s, 3H, acetyl). ^{13}C NMR (101 MHz, D_2O) δ 101.39 (internal C1), 101.36 (internal C1), 101.18 (internal C1), 101.14 (internal C1), 94.72 (β-C1), 90.32 (α-C1), 79.46, 78.96, 78.72, 78.69, 75.79, 74.49, 74.41, 73.31, 72.36, 72.00, 71.95, 69.89, 69.55, 69.11, 60.39, 60.00, 59.84, 55.97, 55.46, 54.95, 54.90, 53.53, 23.14, 22.04, 21.98, 21.74; m/z (HRMS+) 831.3386 [M + H]$^+$ ($C_{32}H_{55}N_4O_{21}$ requires 831.3359).

RP-HPLC of N_4 (ELSD trace, Method A3, t_R = 28.5 min: β-anomer, 30.3 min: α-anomer)

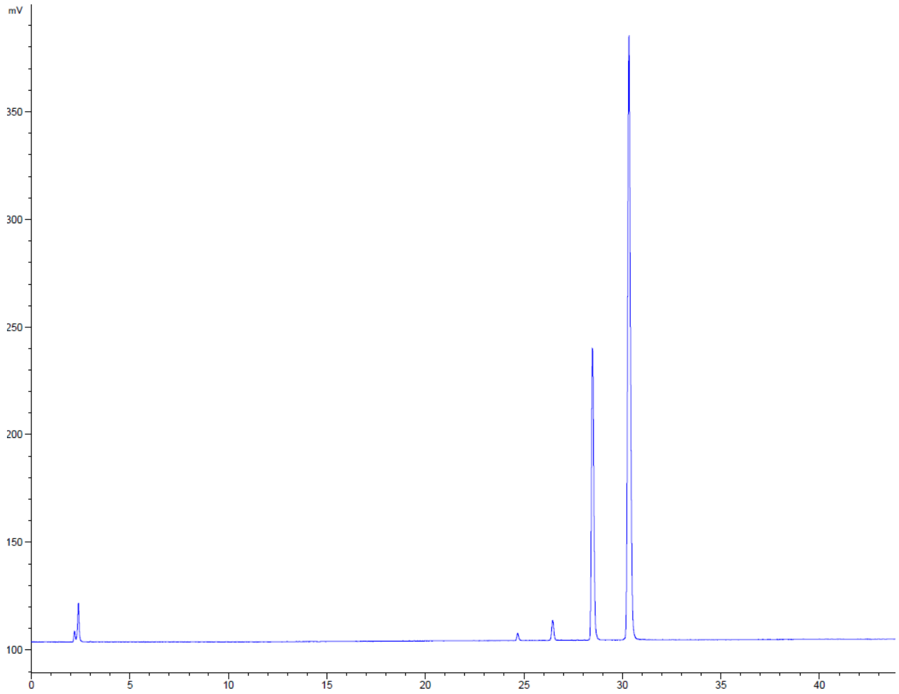

^{1}H NMR of N_4 (400 MHz, D_2O)

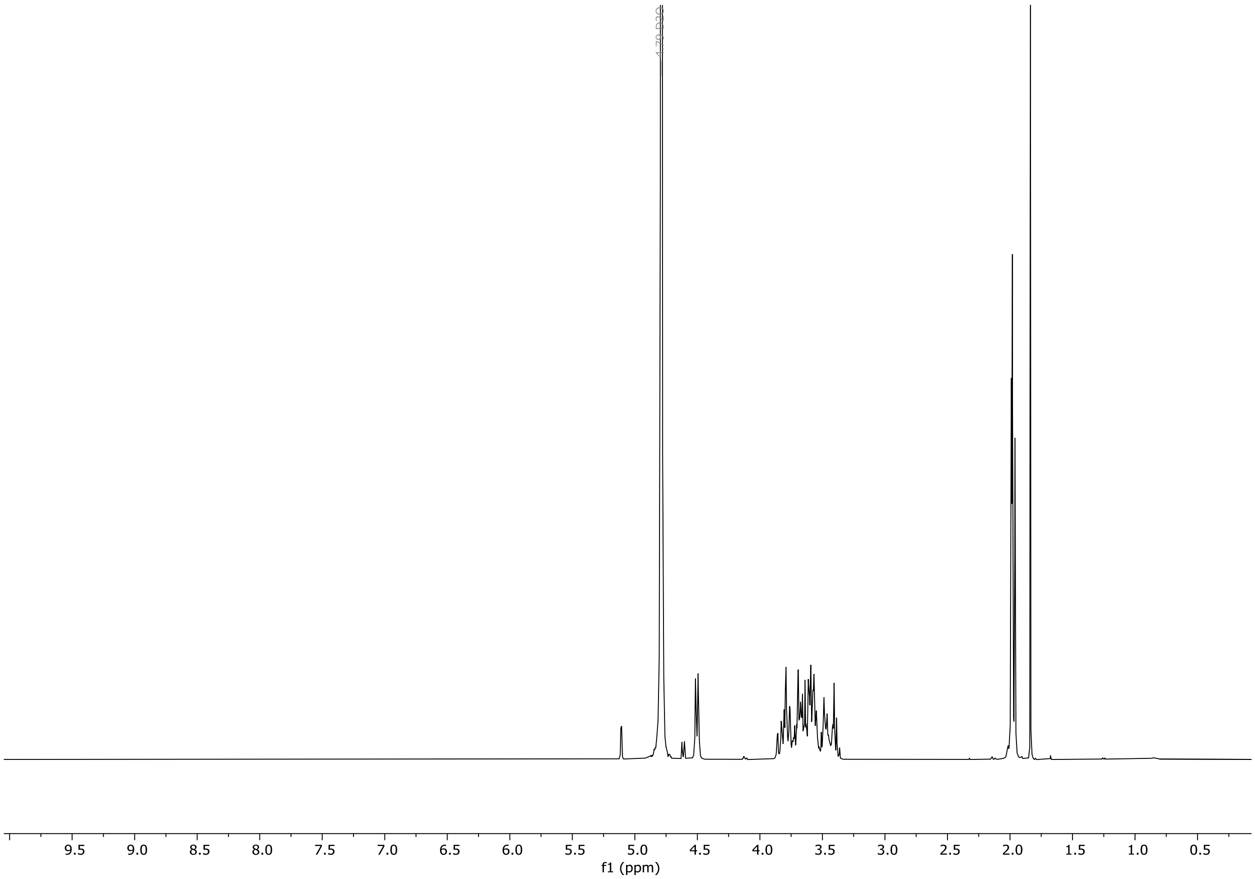

^{13}C NMR of N_4 (151 MHz, D_2O)

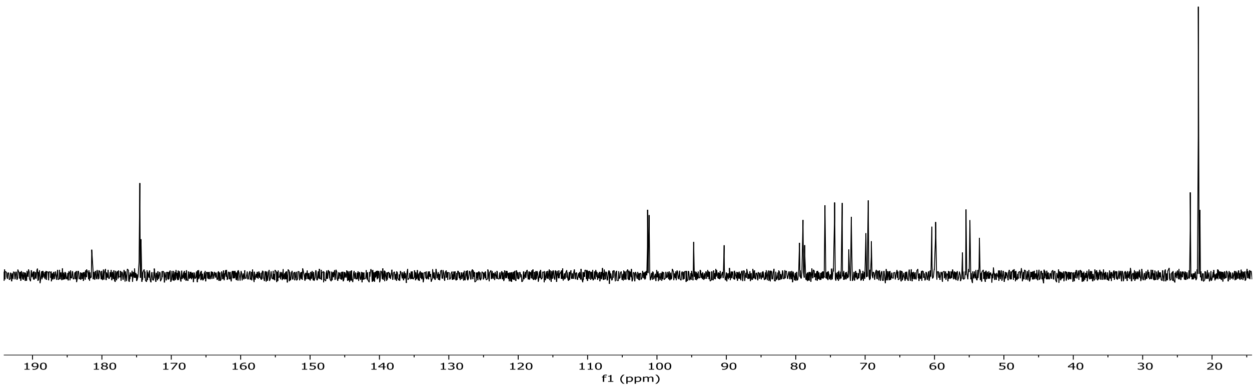

HSQC NMR of N_4 (D_2O)

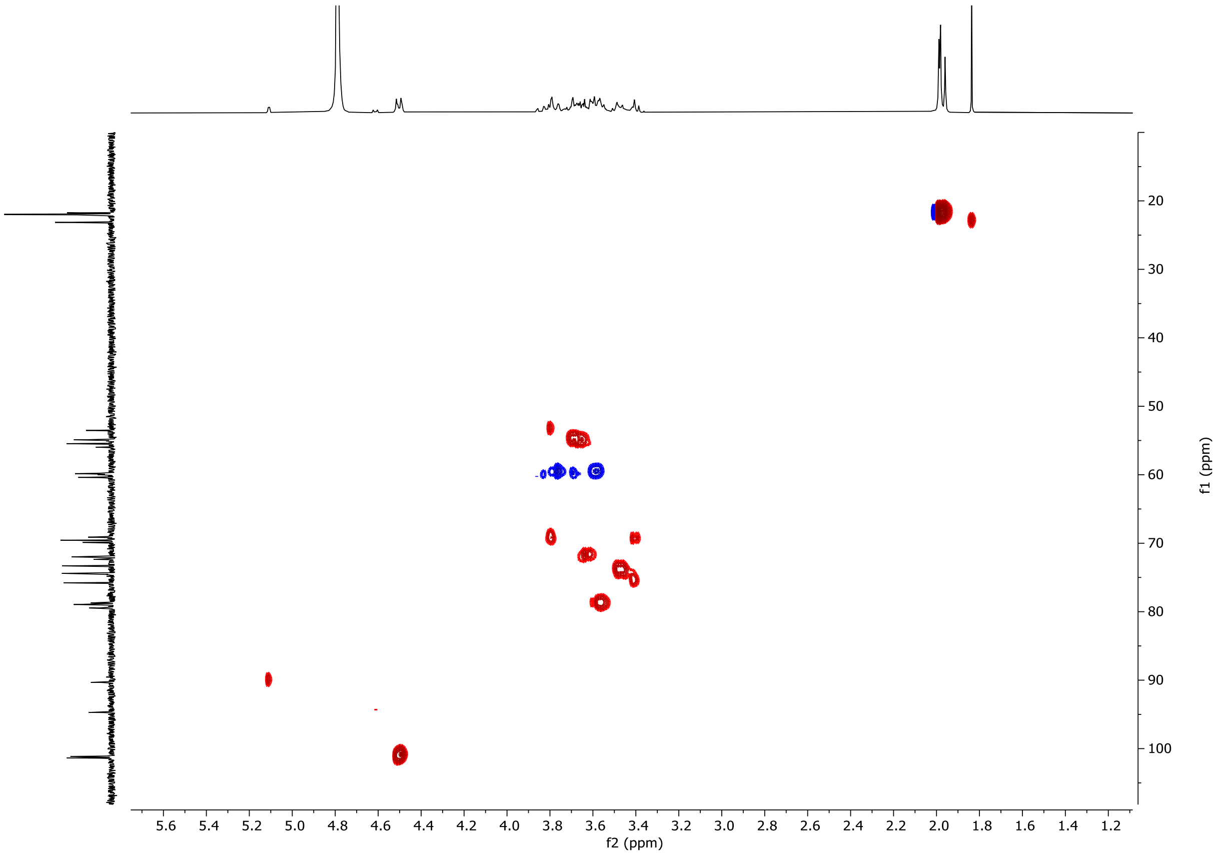

3.8 **Synthesis of N_7**

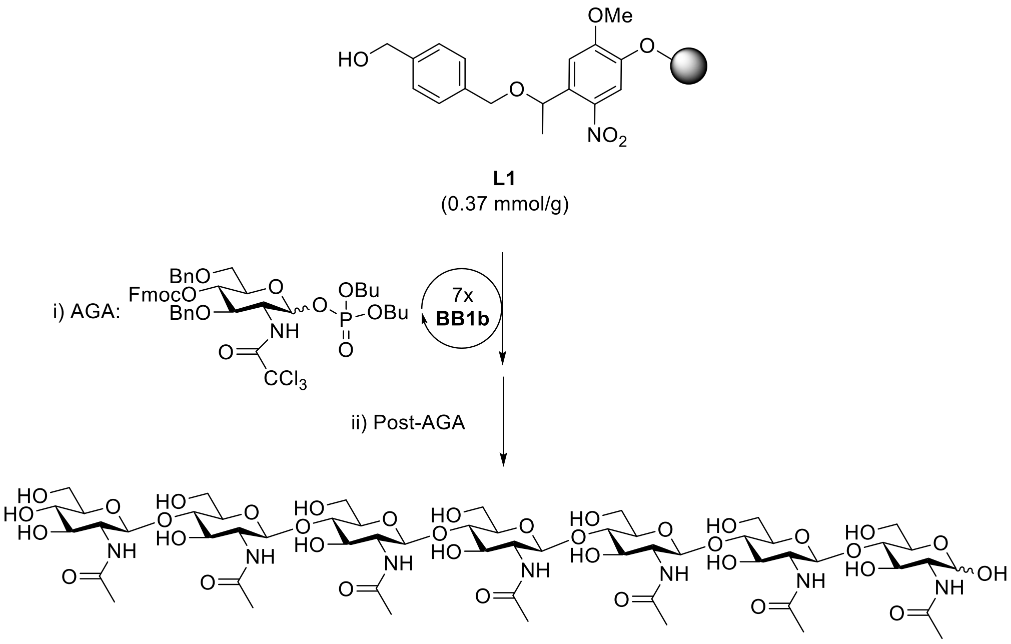

N_7

Step	Modules		Notes
AGA	**L1**	**A**	**L1** swelling
	(**BB1b**)x7	(**B**, **C**, **D**, **E**)x7	**C**: (**BB1b**, -35 °C for 5 min, -10 °C for 40 min)
Post-AGA		**F**, **G**, **H**, **I**	**F**: (16 h)
			**H**: (overnight)
			**I**: (Method A2)

Automated synthesis, global deprotection, and purification afforded compound **N_7** as a white solid (0.9 mg, 5% overall yield).

Analytical data for **N_7**: ^{1}H NMR (400 MHz, D_2O) δ 5.15 (d, *J* = 2.4 Hz, 0.5H, α-H1), 4.65 (d, *J* = 7.9 Hz, 0.5H, β-H1), 4.58 – 4.49 (m, 6H, internal H1), 3.92 – 3.78 (m, 8H), 3.78 – 3.56 (m, 22H), 3.56 – 3.38 (m, 12H), 2.03 (s, 3H, acetyl), 2.02 (s, 15H, acetyls), 2.00 (s, 3H, acetyl).
m/z (HRMS+) 1440.590 $[M + H]^+$ ($C_{56}H_{94}N_7O_{36}$ requires 1440.574).

RP-HPLC of N_7 (ELSD trace, Method A4, t_R = 29.6 min: β-anomer, 30.6 min: α-anomer)

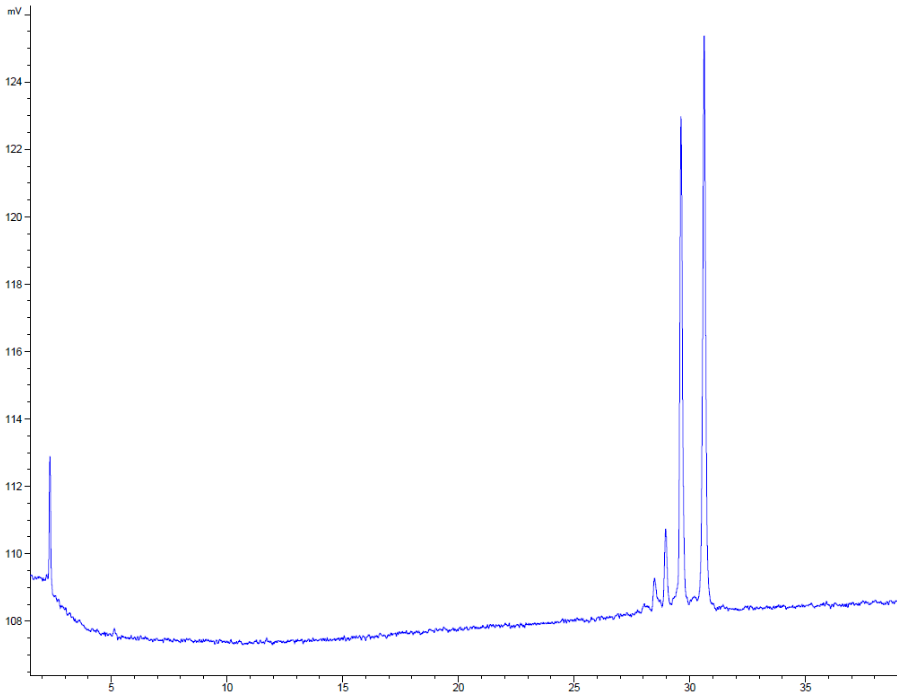

^1H NMR of N_7 (400 MHz, D_2O)

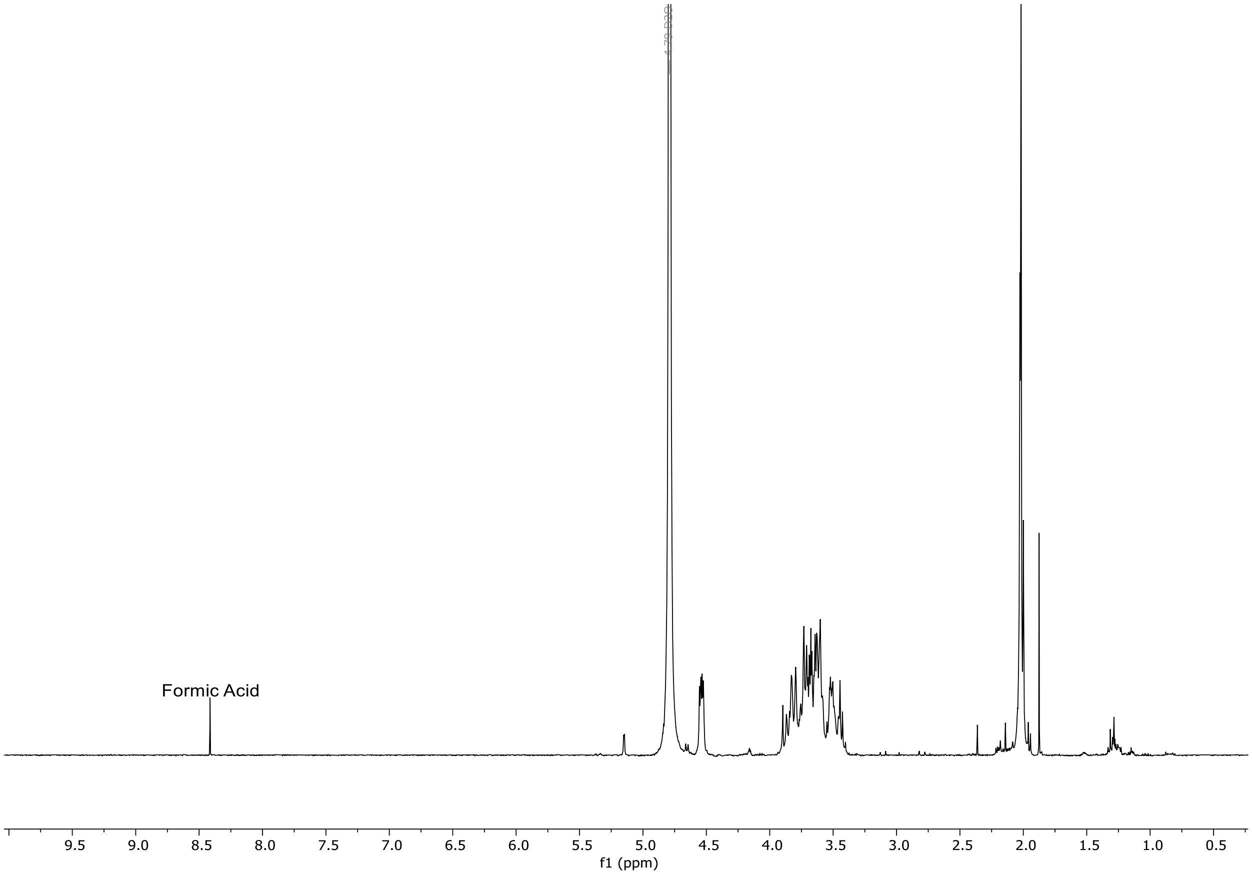

HSQC NMR of N_7 (D_2O)

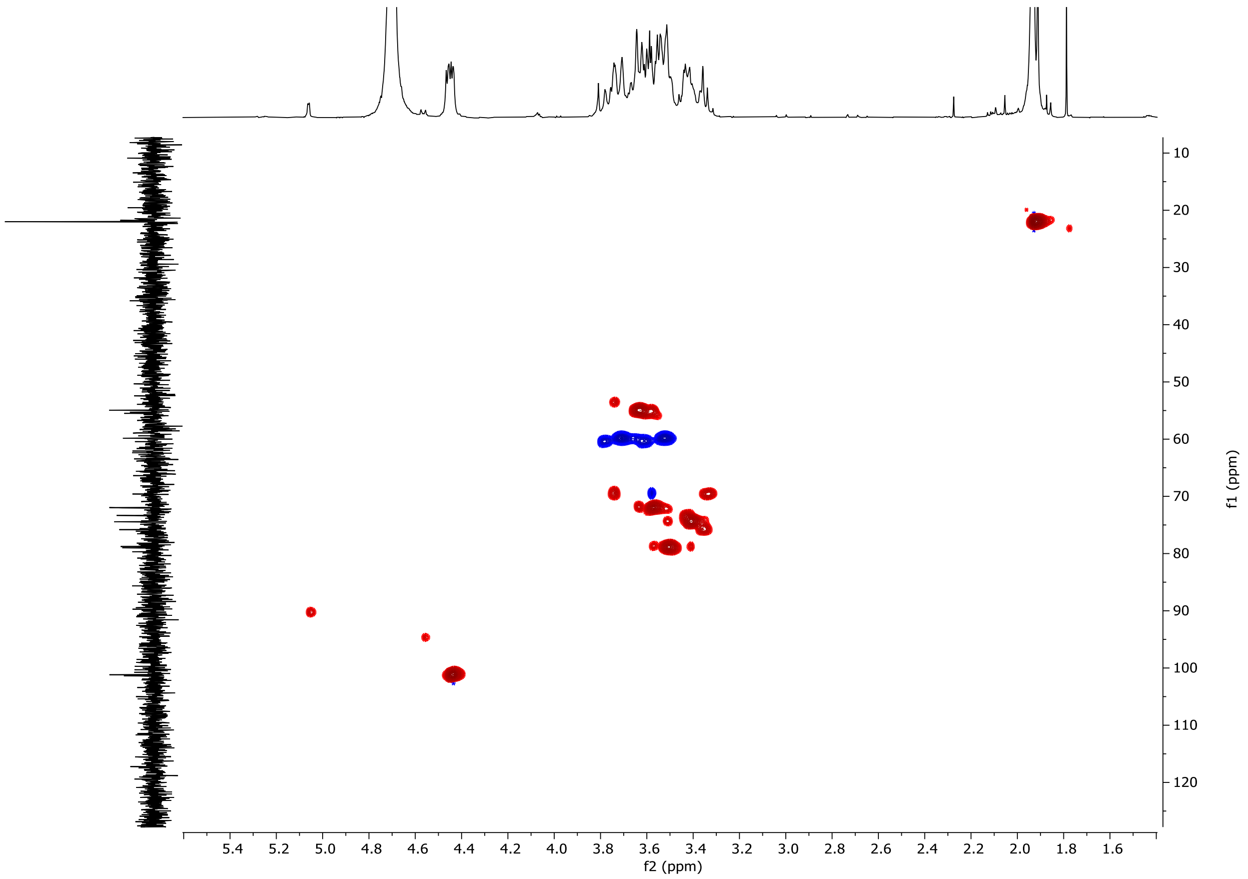

4 Oligosaccharides self-assembly

4.1 X-ray diffraction analysis

X-ray diffraction experiments were carried out using a D8 Avance diffractometer (Bruker) in reflection mode with monochromatic Cu Kα radiation (λ= 1.5418 Å) generated at 40 kV and 40 mA (Siemens X-ray tube KFL CU 2K). The scans were performed in the scattering angle range between 4°and 40° with a step of 0.02°and an accumulation time of 6 or 10 s. Raw XRD profiles were corrected by subtraction of the sample holder signal, smoothing and baseline correction. The oligosaccharide samples were lyophilized prior to XRD measurement.

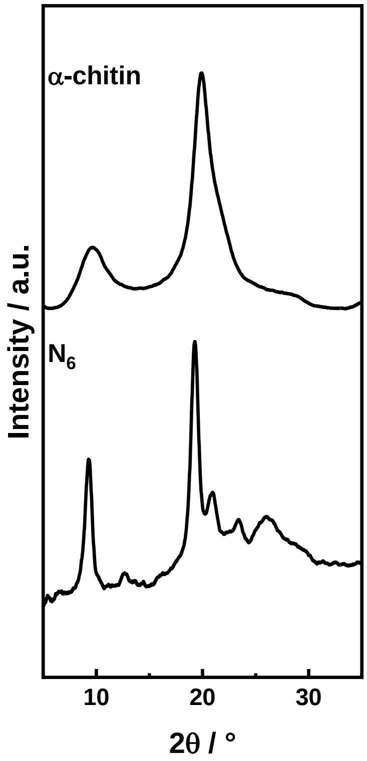

Figure S2 – XRD profile obtained for N_6 and α-chitin.

4.2 Transmission electron microscopy imaging

Transmission electron microscopy (TEM) was performed using a JEOL JEM F200 (Jeol, Japan) (S)TEM equipped with a field emission gun and a TVIPS TemCam-F216 (2k x 2k) camera. The microscope was operated at 80 kV and a condenser aperture with a diameter of 200 µm was used. For specimen preparation, drops (5 µL) of solvent suspensions of samples were deposited on glow-discharged carbon-coated copper grids (Plano GmbH, Germany). The grid was left to dry for 1 h before imaging.

For cryo TEM, a droplet (3 µL) of the suspension of $\mathbf{N_6}$ in isopropanol was deposited on a glow-discharged lacey carbon copper grids. The grid was then blotted with filter paper and subsequently vitrified in liquid ethane using a Leica EM GP plunge freezer (Leica microsystems, Germany) (-180 °C). The frozen grid was then transered to a Gatan Elsa side-entry cryo transfer holder (AMETEK). Cryo TEM observation was performed on a JEM 2100Plus transmission electron microscope (Jeol, Japan) operated an accelerating voltage of 200 kV. The images were recorded on a RIO16 CMOS detector (AMETEK).

To control and monitor humidity and temperature a HUMIgen-04 (Dr. Wernecke Feuchtemesstechnik) was used. The amount of water needed to create a certain relative humidity was calculated according to literature.[6] An ambient airflow of 80 L/h was chosen to minimize the influence on the evaporation process. A modified chamber with external temperature and humidity sensor ensured a stable and homogenous atmosphere throughout the entire experimental setup. To ensure acclimatization of grid and tweezer the chamber was flooded for 30 min before spotting.

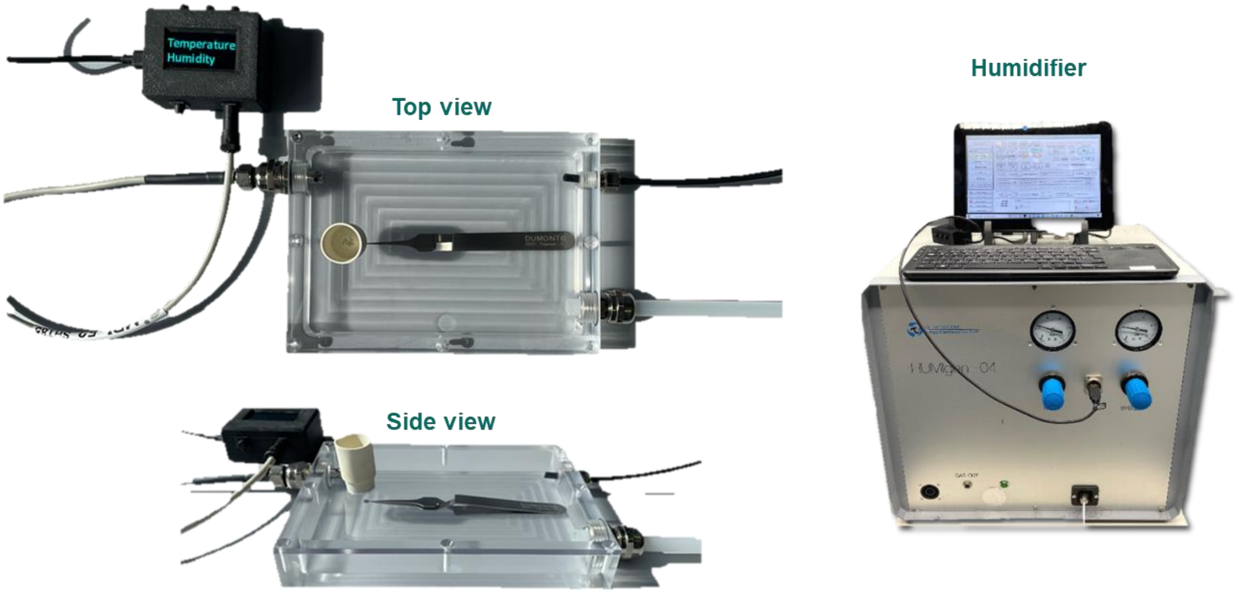

Figure S3 – Experimental setup for the controlled humidity experiments. The humidity chamber allowed for full control over temperature and humidity during the spotting process.

4.2.1 N_6 in H_2O

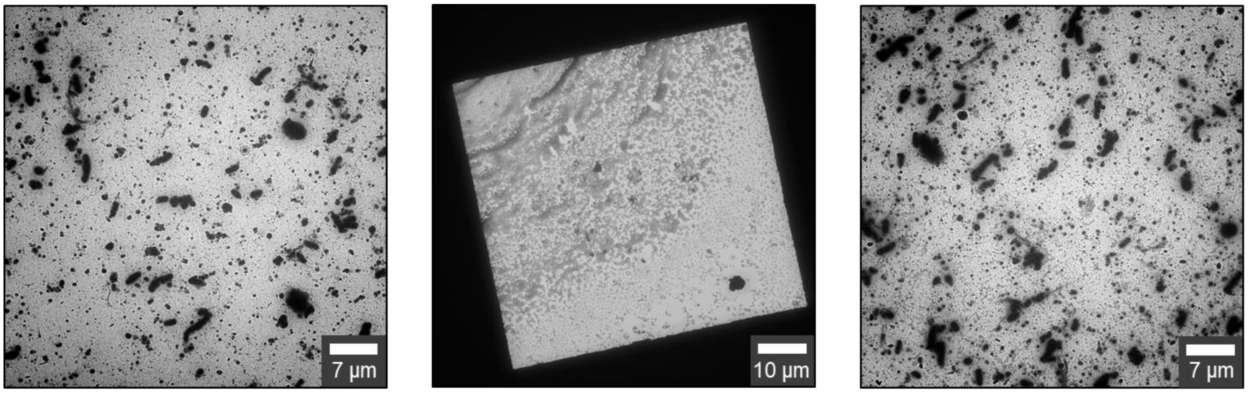

Figure S4 – Representative TEM images of $\mathbf{N_6}$ obtained from aqueous suspension (1.0 mg/mL).

4.2.2 N_6 in hexafluoroisopropanol

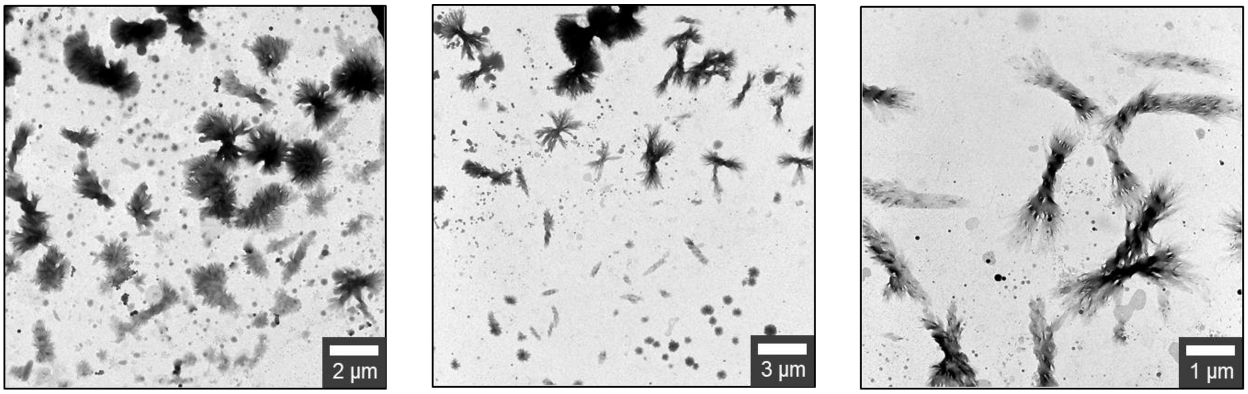

Figure S5 – Representative TEM images of $\mathbf{N_6}$ obtained from suspension in HFIP (0.1 mg/mL).

4.2.3 N_6 in methanol

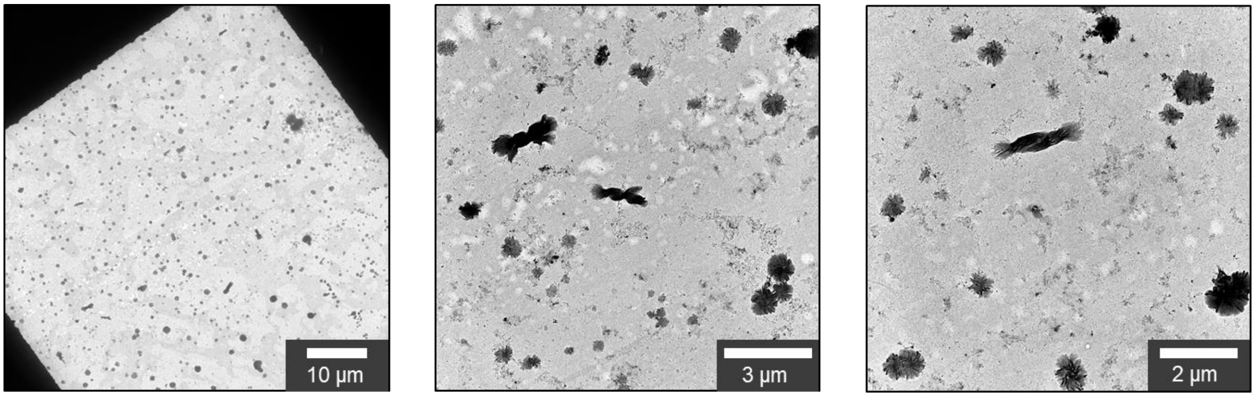

Figure S6 – Representative TEM images of $\mathbf{N_6}$ obtained from suspension in methanol (0.1 mg/mL).

4.2.4 N_6 in ethanol

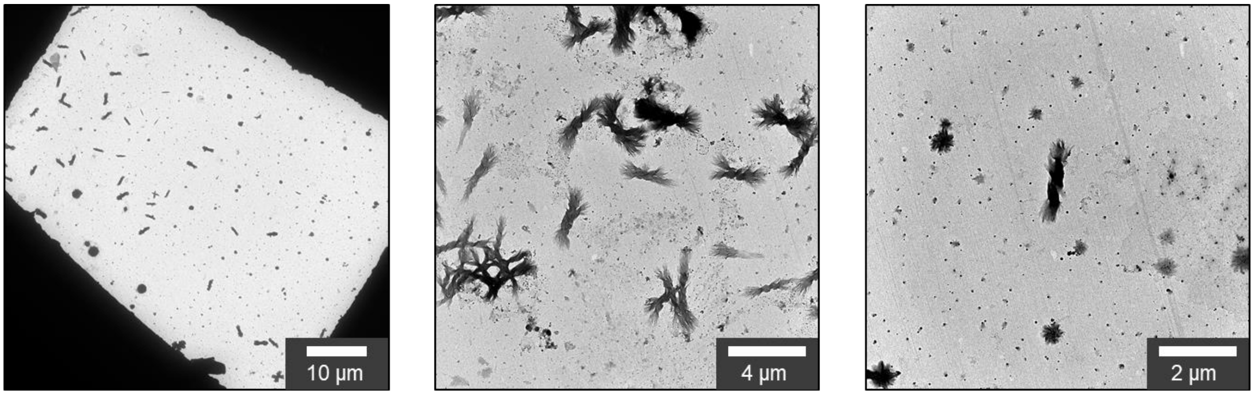

Figure S7 – Representative TEM images of $\mathbf{N_6}$ obtained from suspension in ethanol (0.1 mg/mL).

4.2.5 N_6 in isopropanol

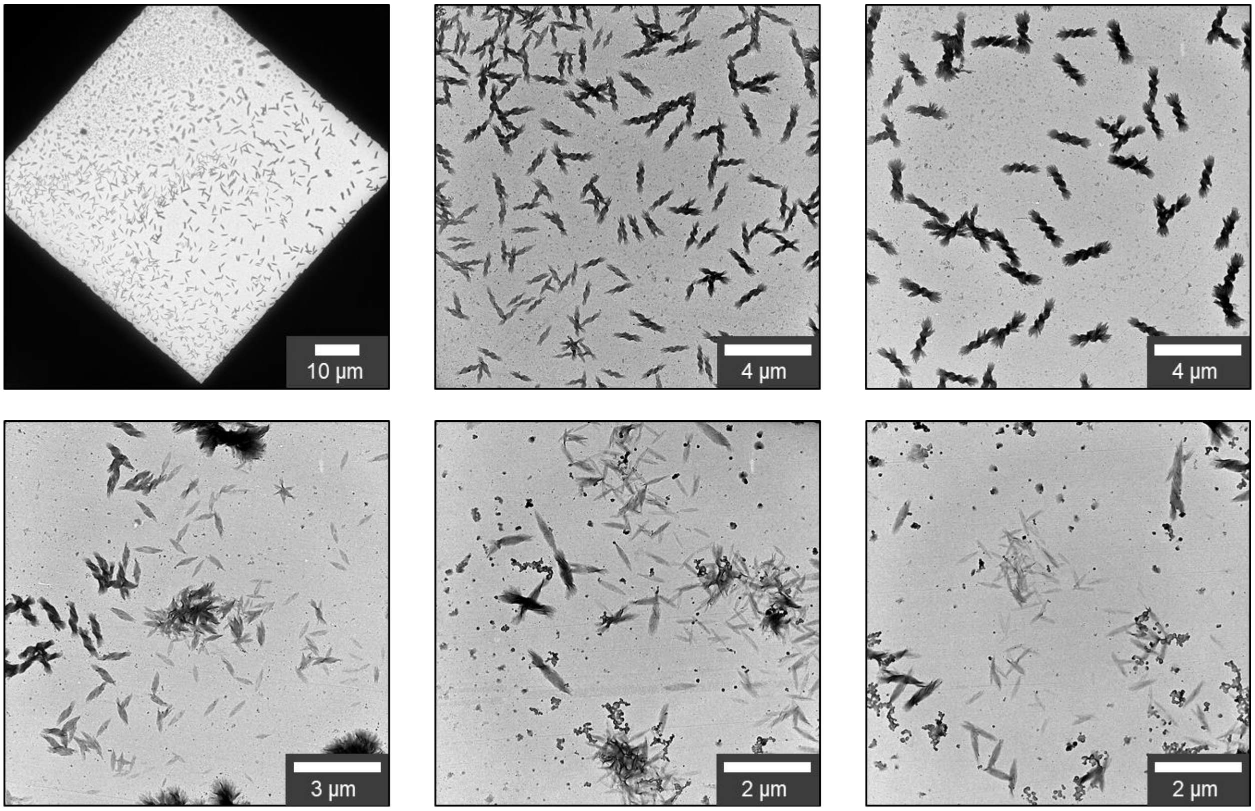

Figure S8 – Representative TEM images of $\mathbf{N_6}$ obtained from suspension in isopropanol (0.1 mg/mL). **Top:** Chiral assemblies. **Bottom:** Single platelets.

4.2.6 Commercial N_6 in isopropanol

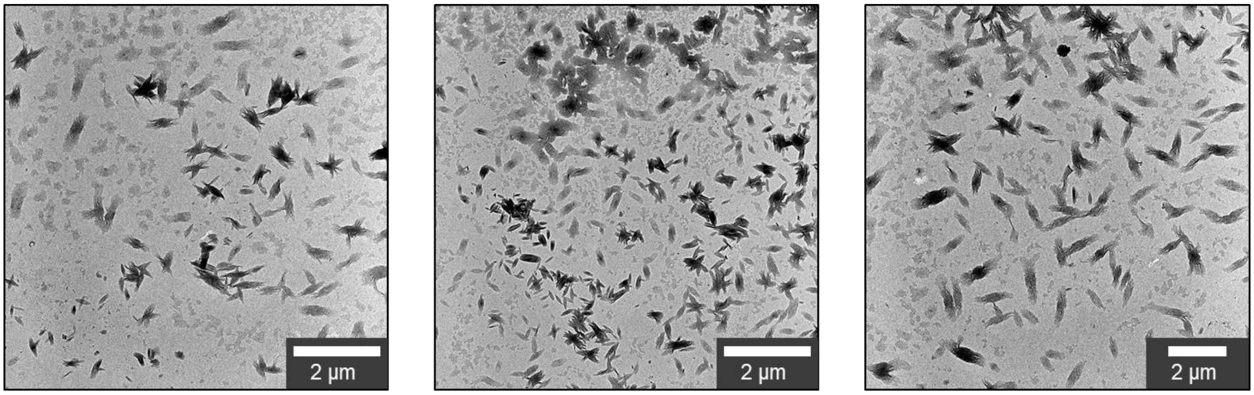

Figure S9 – Representative TEM images of commercially available $\mathbf{N_6}$ obtained from suspension in isopropanol (0.5 mg/mL).

4.2.7 N_4 in isopropanol

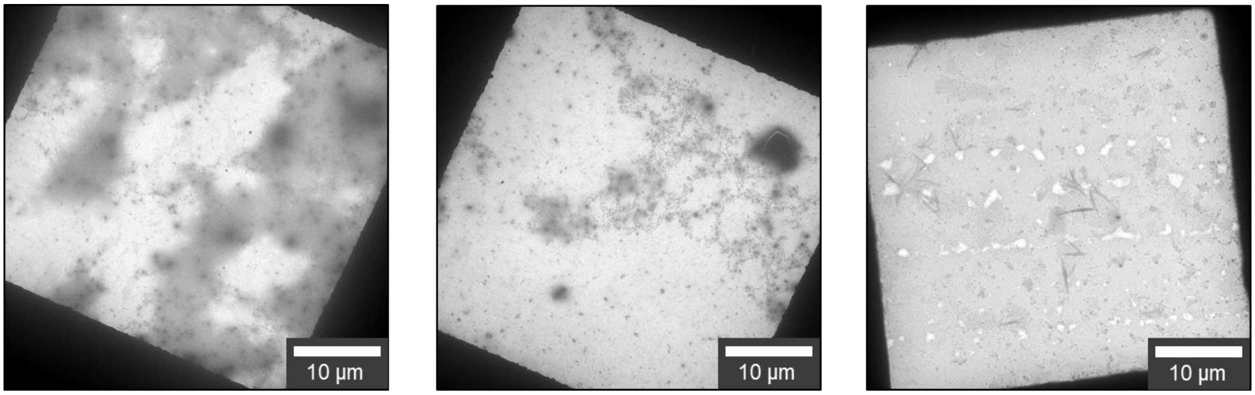

Figure S10 – Representative TEM images of $\mathbf{N_4}$ obtained from suspension in isopropanol (1.0 mg/mL).

4.2.8 N_7 in isopropanol

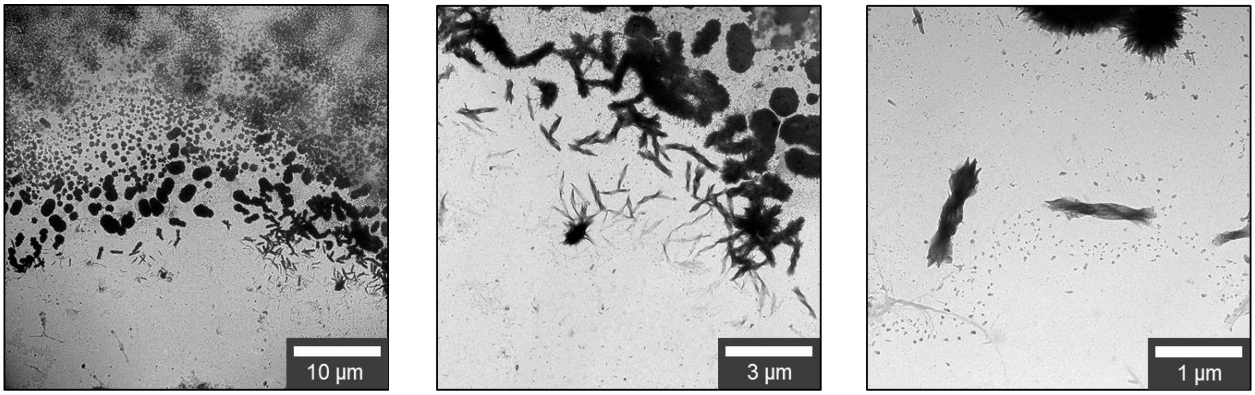

Figure S11 – Representative TEM images of $\mathbf{N_7}$ obtained from suspension in isopropanol (1.0 mg/mL).

4.2.9 Degree of polymerization

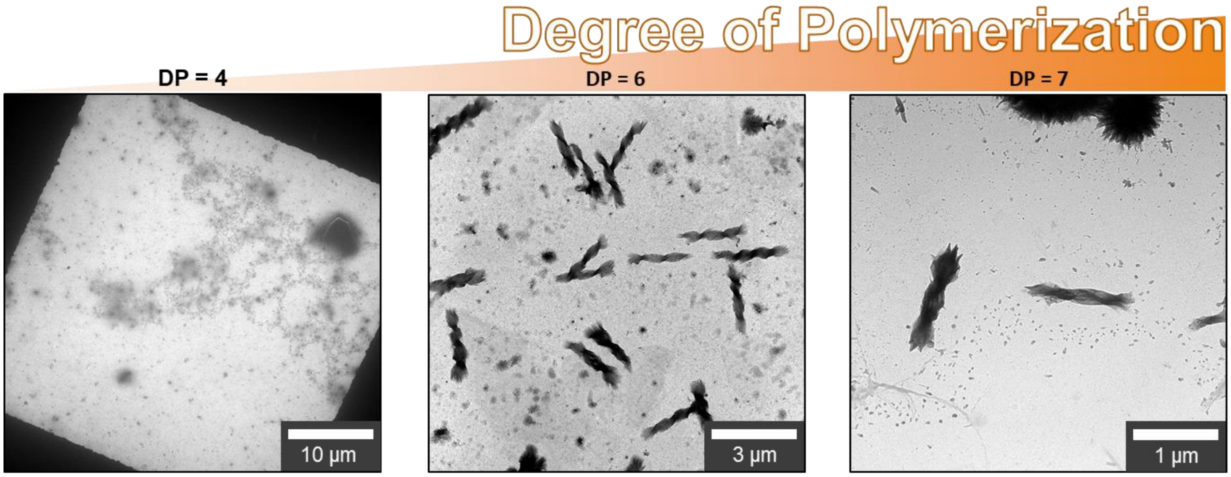

Figure S12 – Representative TEM images of $\mathbf{N_4}$ (left) $\mathbf{N_6}$ (middle) and $\mathbf{N_7}$ (right) obtained from suspension in isopropanol.

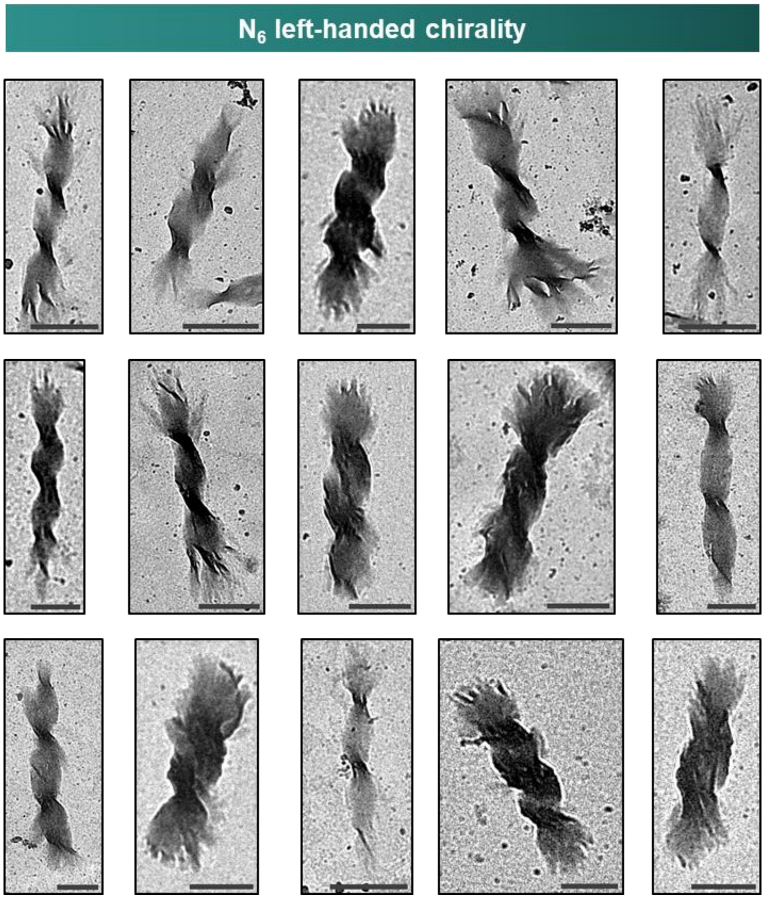

Figure S13 – Excerpts of TEM images of $\mathbf{N_6}$ twisted assemblies obtained from suspension in isopropanol showing left handed twists. Scale bar 500 nm.

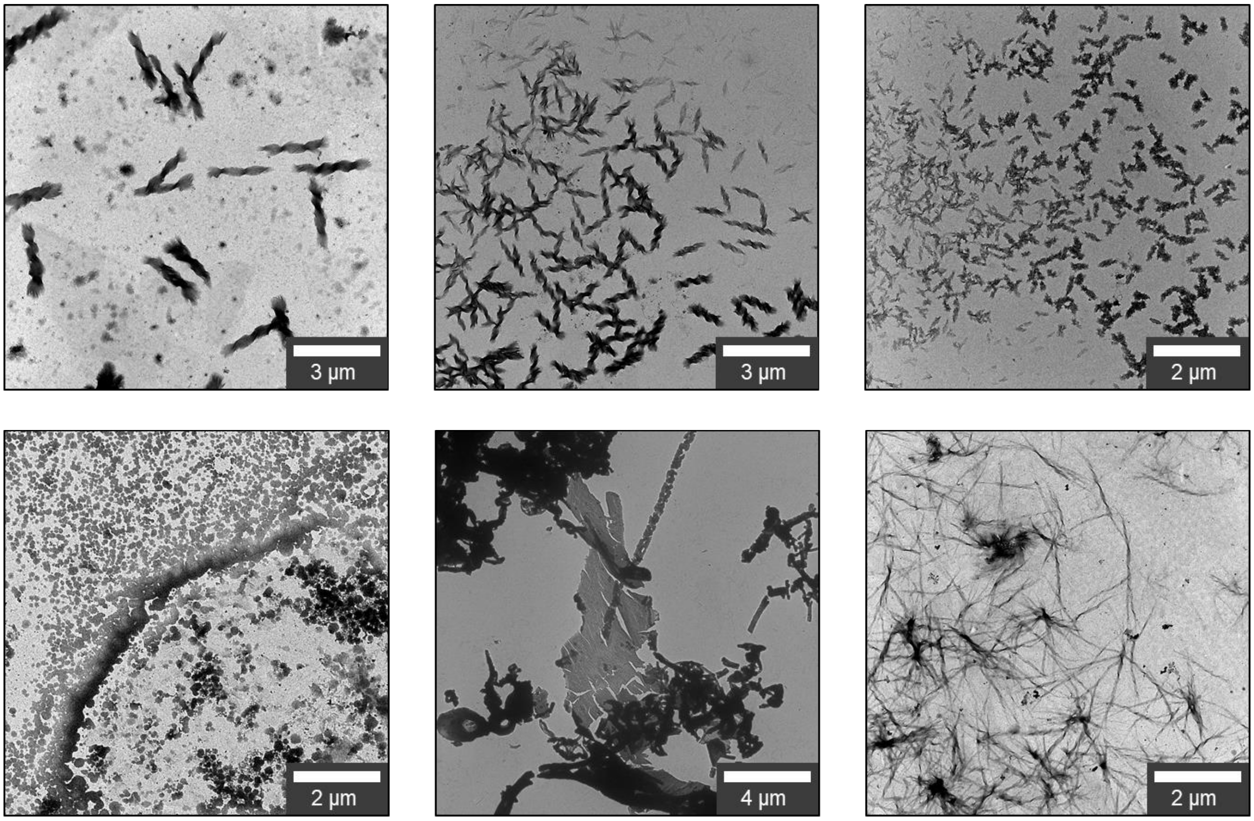

Figure S14 – Representative TEM images of $\mathbf{N_6}$ obtained from suspension in isopropanol (0.5 mg/mL) showing different kind of assemblies. **Top:** Defined (**left**, **middle**) and less defined (**right**) chiral twisted assemblies. **Bottom:** Amorphous aggregates (**left**), cramped aggregates (**middle**) and long thin fibrils (**right**).

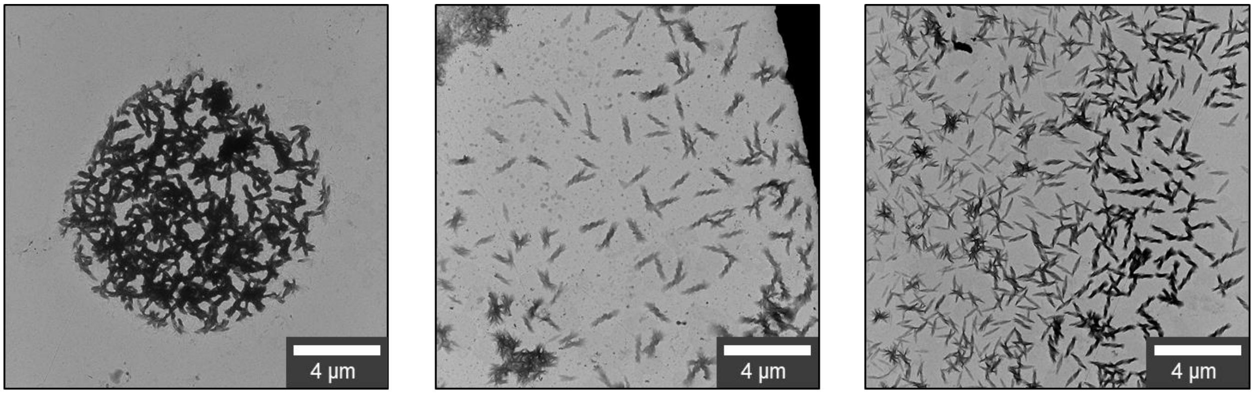

Figure S15 – Representative TEM images of $\mathbf{N_6}$ obtained from suspension in isopropanol (0.5 mg/mL) with variations of glow discharge strength. **Left:** No glow discharged grid. **Middle:** Normal glow discharged grid (15 mA for 15 s). **Right:** Strong glow discharged grid (20 mA for 15 s).

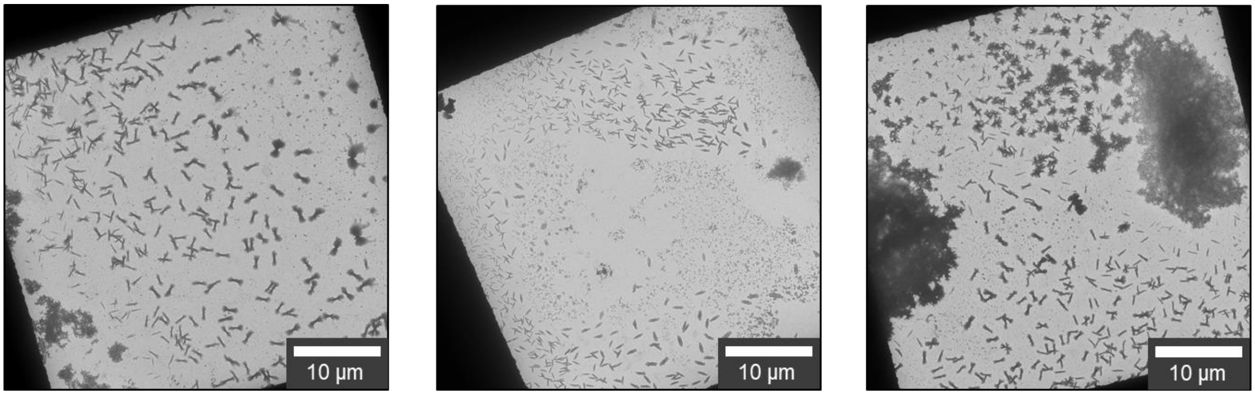

Figure S16 – Representative TEM images of $\mathbf{N_6}$ obtained from suspension in isopropanol (0.5 mg/mL) with different aging time. **Left:** 1 day. **Middle:** 4 days. **Right:** 6 days.

4.2.10 N6 with controlled humidity

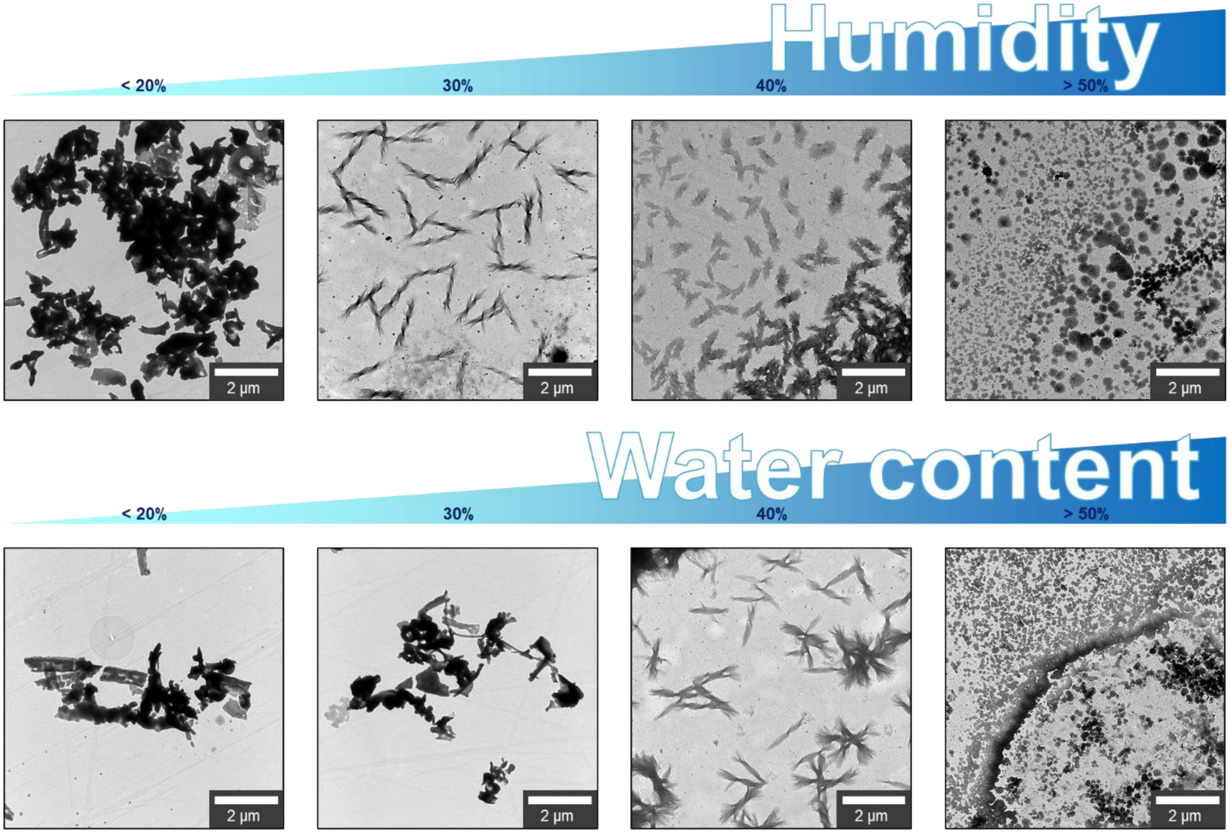

Figure S17 – **Top:** Representative TEM images of $\mathbf{N_6}$ obtained from suspension in isopropanol (0.5 mg/mL) with changing humidity. **Bottom:** Representative TEM images of $\mathbf{N_6}$ obtained from suspension in a mixture of water and isopropanol (0.5 mg/mL) with ratio.

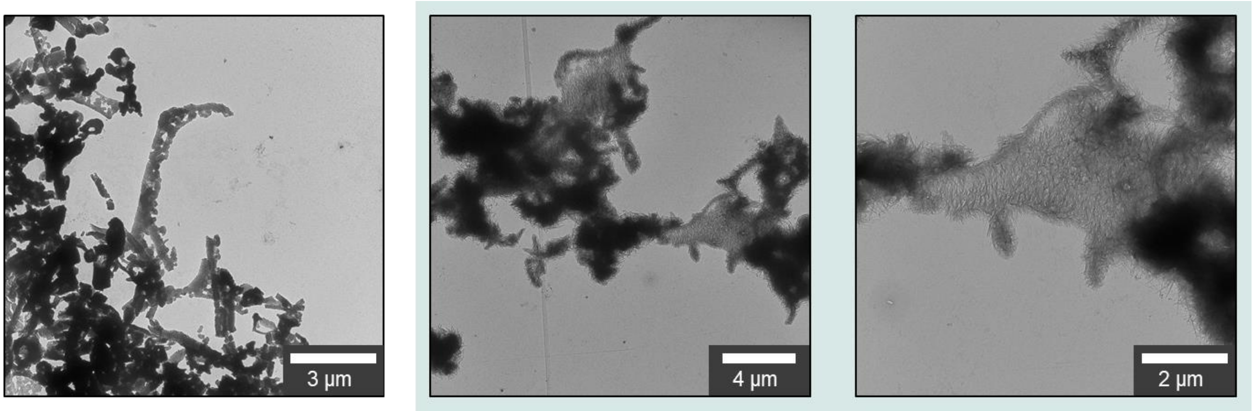

Figure S18 – Representative TEM images of $\mathbf{N_6}$ obtained from suspension in isopropanol (0.5 mg/mL) before and after humidity treatment. **Left:** Cramped aggregates obtained under dry spotting conditions (humidity < 20%). **Blue background:** The same sample after humidity treatment (2 h incubation in a humidity chamber with 100% humidity).

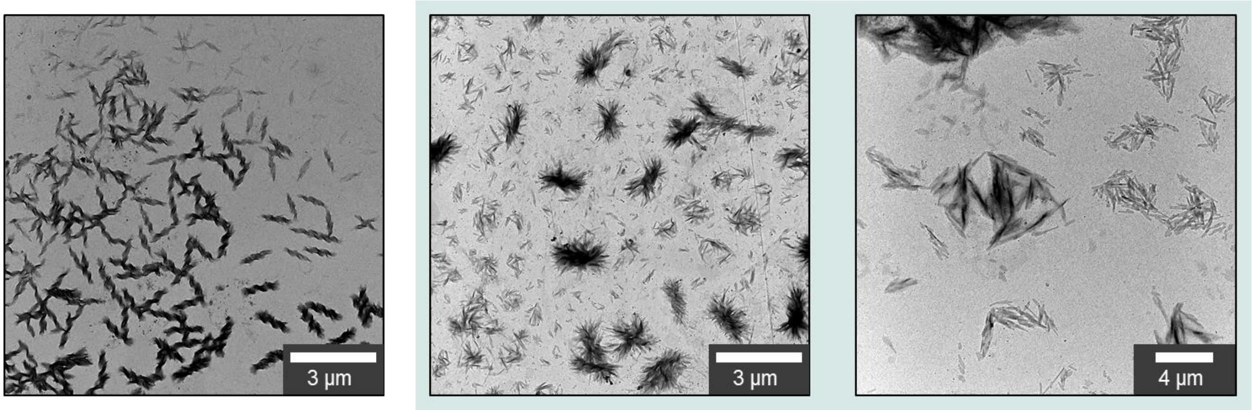

Figure S19 – Representative TEM images of $\mathbf{N_6}$ obtained from suspension in isopropanol (0.5 mg/mL) before and after humidity treatment. **Left:** Twisted assemblies obtained under ambient spotting conditions (humidity = 30%). **Blue background:** The same sample after humidity treatment (2 h incubation in a humidity chamber with 100% humidity).

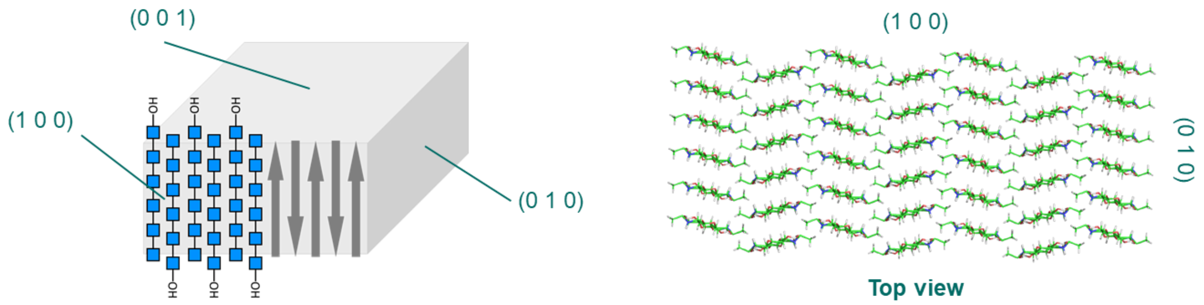

Figure S20 – Illustration of the reconstructed crystal structure of **N$_6$**.

4.2.11 Cryo-TEM of N$_6$ in isopropanol

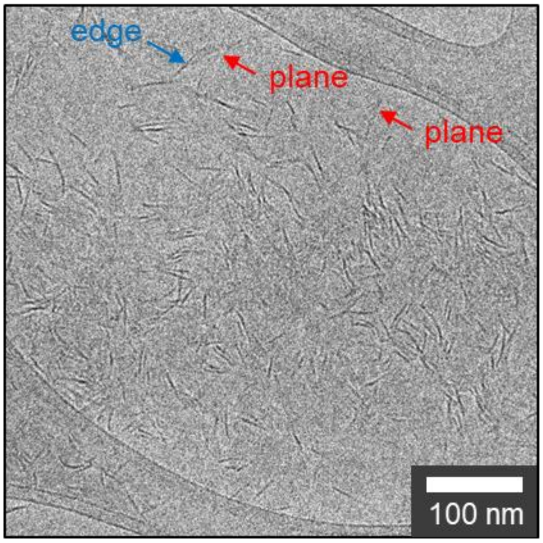

Figure S21 – Cryo-TEM image of **N$_6$** platelets present in solution.

4.3 Atomic force microscopy imaging

Atomic force microscopy was performed with a Jupiter XR AFM (Oxford Instruments) in tapping mode (AC mode) using Arrow NCR tip (42 N/m, 285 kHz, Nano World). The samples were prepared as follows: approximately 0.1 mg of the lyophilized powder was weighed (in a glass or plastic vial), diluted with isopropanol to reach the concentration of 1 mg/mL. For AFM imaging, the solution was further diluted with isopropanol to reach the concentration of 0.1 mg/mL. Drops of aqueous suspensions were deposited on freshly glow-discharged (0.8 mbar, 15 mA for 20 s using air) copper grid and dried at room temperature. AFM images were collected with 2048 x 2048 pixels/frame and analyzed with the AR 18.10.29 processing software (AsylumResearch Analyses Software). To determine the dimensions of the chiral assemblies, the obtained AFM height images were analyzed according to length, width and height of single uncovered aggregates. In total 310 particles were analyzed.

Particle	Number	310
**Length**	1944	± 336 nm
**Width**	484	± 48 nm
**Height**	103	± 15 nm

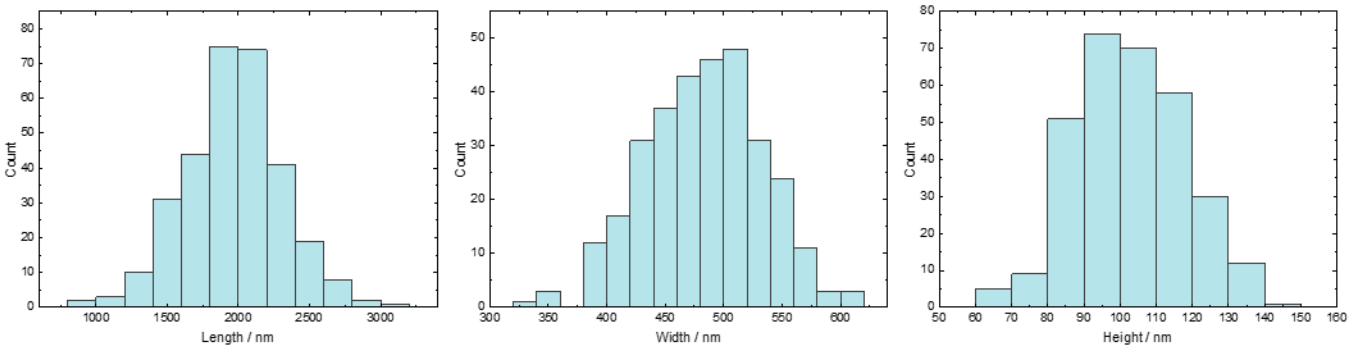

Figure S22 – Histogram of the dimensions of 310 chiral assemblies obtained from the analysis of AFM height images of $\mathbf{N_6}$ in isopropanol (0.5 mg/mL) spotted on a TEM grid.

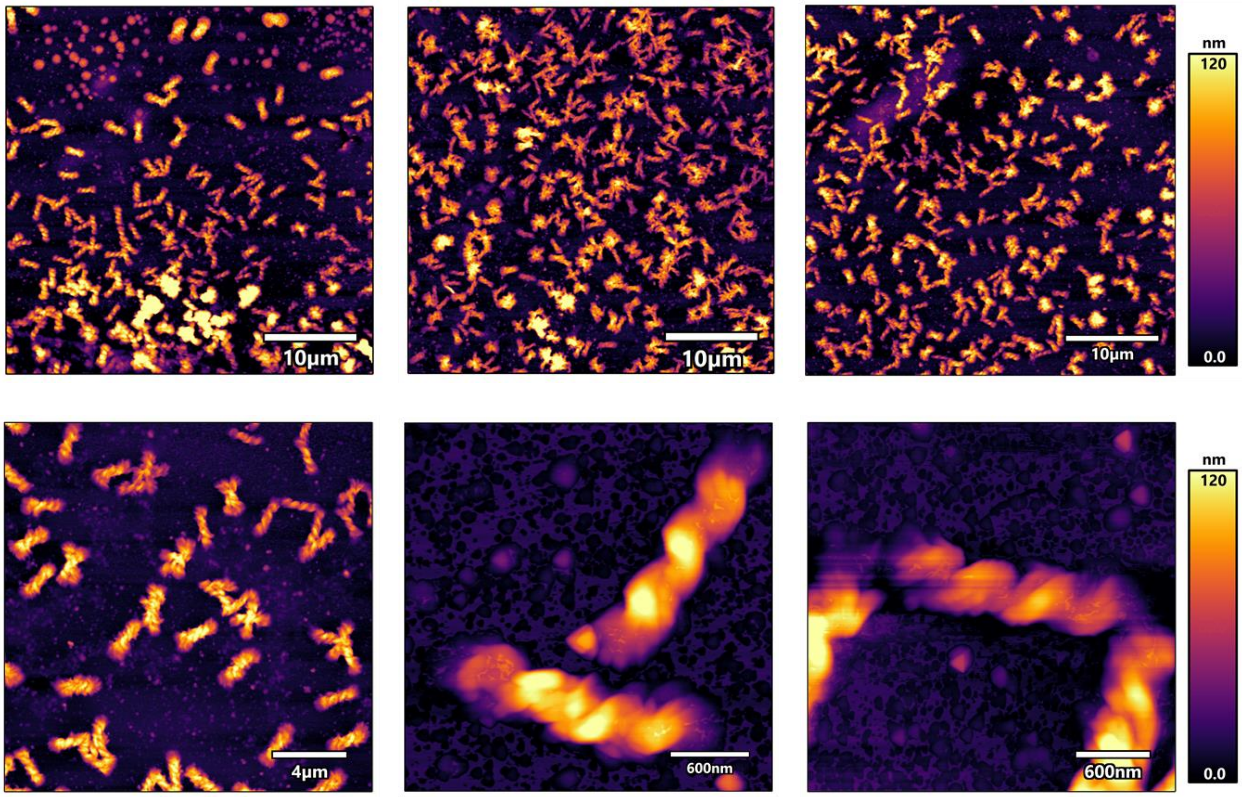

Figure S23 – Representative AFM height images of $\mathbf{N_6}$ obtained from suspension in isopropanol (0.5 mg/mL) spotted on a TEM grid.

4.4 Photo-induced force microscopy (PiFM)

The PiFM measurements were carried out using an atomic force microscope (model VistaScope, Molecular Vista, Inc.). The $\mathbf{N_6}$ assemblies on carbon-coated copper TEM grids were scanned with a gold-coated silicon cantilever. The excitation laser for the PiFM measurements is a LaserTune IR Source from Block Engineering. PiF-IR spectra were recorded from 500 cm^{-1} to 3600 cm^{-1}. The spectra were then smoothed by Savitzky-Golay filter and normalized to the intensity of 2850 cm^{-1}.

5 References

(1) Eller, S.; Collot, M.; Yin, J.; Hahm, H. S.; Seeberger, P. H. Automated solid-phase synthesis of chondroitin sulfate glycosaminoglycans. *Angew. Chem., Int. Ed* **2013**, *52* (22), 5858-5861.

(2) Tyrikos-Ergas, T.; Bordoni, V.; Fittolani, G.; Chaube, M. A.; Grafmüller, A.; Seeberger, P. H.; Delbianco, M. Systematic structural characterization of chitooligosaccharides enabled by Automated Glycan Assembly. *Chem. Eur. J.* **2021**, *27* (7), 2321-2325.

(3) Le Mai Hoang, K.; Pardo-Vargas, A.; Zhu, Y.; Yu, Y.; Loria, M.; Delbianco, M.; Seeberger, P. H. Traceless photolabile linker expedites the chemical synthesis of complex oligosaccharides by automated glycan assembly. *J. Am. Chem. Soc.* **2019**, *141* (22), 9079-9086.

(4) Gude, M.; Ryf, J.; White, P. D. An accurate method for the quantitation of Fmoc-derivatized solid phase supports. *Lett. Pept. Sci.* **2002**, *9* (4-5), 203-206.

(5) Hurevich, M.; Kandasamy, J.; Ponnappa, B. M.; Collot, M.; Kopetzki, D.; McQuade, D. T.; Seeberger, P. H. Continuous photochemical cleavage of linkers for solid-phase synthesis. *Org. Lett.* **2014**, *16* (6), 1794-1797.

(6) Green, D. W.; Southard, M. Z. *Perry's chemical engineers' handbook*; McGraw-Hill Education, 2019.
